# The Impact of Land Abandonment on Species Richness and Abundance in the Mediterranean Basin: A Meta-Analysis

**DOI:** 10.1371/journal.pone.0098355

**Published:** 2014-05-27

**Authors:** Tobias Plieninger, Cang Hui, Mirijam Gaertner, Lynn Huntsinger

**Affiliations:** 1 Department of Geosciences and Natural Resource Management, University of Copenhagen, Frederiksberg, Denmark; 2 Centre for Invasion Biology, Department of Mathematical Sciences, Stellenbosch University, Matieland, South Africa; 3 Mathematical and Physical Biosciences, African Institute for Mathematical Sciences, Cape Town, South Africa; 4 Department of Environmental Science, Policy, and Management, University of California, Berkeley, California, United States of America; National University of Singapore, Singapore

## Abstract

Land abandonment is common in the Mediterranean Basin, a global biodiversity hotspot, but little is known about its impacts on biodiversity. To upscale existing case-study insights to the Pan-Mediterranean level, we conducted a meta-analysis of the effects of land abandonment on plant and animal species richness and abundance in agroforestry, arable land, pastures, and permanent crops of the Mediterranean Basin. In particular, we investigated (1) which taxonomic groups (arthropods, birds, lichen, vascular plants) are more affected by land abandonment; (2) at which spatial and temporal scales the effect of land abandonment on species richness and abundance is pronounced; (3) whether previous land use and current protected area status affect the magnitude of changes in the number and abundance of species; and (4) how prevailing landforms and climate modify the impacts of land abandonment. After identifying 1240 potential studies, 154 cases from 51 studies that offered comparisons of species richness and abundance and had results relevant to our four areas of investigation were selected for meta-analysis. Results are that land abandonment showed slightly increased (effect size  = 0.2109, P<0.0001) plant and animal species richness and abundance overall, though results were heterogeneous, with differences in effect size between taxa, spatial-temporal scales, land uses, landforms, and climate. In conclusion, there is no “one-size-fits-all” conservation approach that applies to the diverse contexts of land abandonment in the Mediterranean Basin. Instead, conservation policies should strive to increase awareness of this heterogeneity and the potential trade-offs after abandonment. The strong role of factors at the farm and landscape scales that was revealed by the analysis indicates that purposeful management at these scales can have a powerful impact on biodiversity.

## Introduction

Increasing competition for land is one of the most significant processes of global environmental change [1], [2]. Though obscured by the attention given to global land scarcity, the phenomenon of land abandonment – change towards termination of crop cultivation or livestock grazing [3] – is equally on the rise [4], [5]. Cropland abandonment has affected an estimated 1.47 million km^2^ worldwide from the 1700s to 1992 [6]. Agricultural abandonment has been substantial throughout the 20^th^ century in North America, the former Soviet Union and Southern Asia, followed by Europe, South America and China since the 1960s [7]. A set of underlying and proximate ecological (e.g. declining soil fertility), social (rural depopulation) and economic (e.g. globalization of agro-commodity markets, declining farm profitability) drivers determine the patterns and processes of land abandonment, usually through interaction at various spatial and temporal scales [8]. Land abandonment has a range of consequences for the provision of ecosystem processes, including functions and services that are not well-understood and often context-specific, for example wildfire frequency and intensity, nutrient cycling, carbon sequestration, cultural landscape values and water balance [3]. Here we conduct a meta-analysis of the literature to examine the consequences of land abandonment in the Mediterranean Basin.

### Consequences of Land Abandonment

Two fundamentally different biodiversity consequences are possible: On the one hand, land abandonment may contribute to “passive landscape restoration” [9] or “rewilding” [10], thus facilitating the restoration of natural ecosystem processes and reducing direct human influence on landscapes. Several studies confirm that, for example, woodland bird and large mammal populations benefit from large-scale land abandonment (see [11] and references therein). On the other hand, abandonment of agricultural landscapes may threaten farmland biodiversity, in particular functional diversity [12] associated with anthropogenic landscapes of high nature value. “High nature value farming” is a predominantly European concept that recognizes that the conservation of biodiversity in some settings depends on the continuation of low-intensity farming systems [13]–[15]. Processes induced by abandonment of agricultural uses that may threaten local biodiversity include habitat loss, decrease in habitat patchiness, competitive exclusion, invasions of non-native plants, litter accumulation, increased predation, and increased wildfires [3].

Put into a larger perspective, the dispute between “rewilding” and “high nature value farming” advocates reflects the ongoing scholarly debate of whether biodiversity conservation should pursue “land sparing” (embracing “rewilded” ecosystems) or “land sharing” (calling for “high nature value” farming) [16], [17]. Trajectories of land abandonment are accompanied by considerable societal trade-offs, not only between the different kinds of biodiversity that are supported or degraded, but also between ecosystem functions and services such as aesthetic values, carbon sequestration, or wildfire regimes in landscapes [5]. Despite the implications of these diverging views for conservation, the biodiversity impacts of land abandonment have only started to be assessed beyond local-scale case studies [18], [19].

### Objective

The Mediterranean Basin is one of the original 25 global biodiversity hotspots [20], exhibiting high levels of plant and animal richness and endemism [11], [21], [22]. There are numerous case studies on the impacts of land abandonment. To upscale these local-scale case study insights, we performed a meta-analysis focusing on the effects of land abandonment on plant and animal species richness and abundance in agroforestry, arable land, pastures, and permanent crops of the Mediterranean Basin. Based on a previously developed review protocol [23], we investigated (1) which taxonomic groups (arthropods, birds, lichen, vascular plants) are more affected by land abandonment; (2) at which spatial and temporal scales the effect of land abandonment on species richness and abundance is pronounced; (3) whether previous land use and current protected area status affect the magnitude of changes in the number and abundance of species; and (4) how prevailing landforms (mountain vs. lowland areas) and climate modify the impacts of land abandonment. Mediterranean-type environments are particularly suitable for meta-analysis, as they vary less in climate, disturbance regimes, and further key aspects than other biome types [24]. Previous reviews have covered land abandonment [3], [8], [18], but did not perform formal meta-analyses and/or did not cover a particular biodiversity hotspot. Our intention is to identify knowledge gaps and to inform conservation policy.

## Materials and Methods

### Study Area: Mediterranean Basin

The Mediterranean Basin is one of the world's regions where land abandonment is prevalent [25], [26], especially in upland areas [27]. Precise data on land abandonment are not available, but FAO forest statistics indicate that most of the abandoned Mediterranean farmland is in the European Union member countries, Israel, Turkey and Algeria [28]. Old fields have always been part of a dynamic equilibrium in Mediterranean landscapes, but permanent land abandonment has increased throughout the 20^th^ century [29]. In most northern Mediterranean countries forest cover has increased by about 2% per annum [11].

Modernization of agricultural production in fertile lowland areas and a population exodus from rural areas to urban centers have been the most decisive drivers of Mediterranean land abandonment [30]–[33]. Agricultural land uses are generally given up when farming fails to adjust to changed economic conditions. The physical constraints of soils, topography, climate, and remoteness limit the options for adaptation to more intensive, mechanized, and profitable farming techniques on the marginal lands of the Mediterranean Basin. Agricultural policies have further accelerated the concentration of agricultural activities on more fertile and accessible land and the abandonment of marginal lands, though some more recent rural development policies have mitigated this trend [27], [34], [35].

The rich biodiversity of the Mediterranean Basin is the consequence of a particular biogeography, geological history, landscape ecology, and human history. Most notably, human land uses have shaped ecosystems for more than 10,000 years and have enhanced biological and landscape diversity [29], [31]. Given that the Mediterranean biome has been predicted to experience the greatest proportional change in biodiversity by 2100, mainly through land use and climate change [36], questions about the impacts of land abandonment on biodiversity are critical.

### Study Selection

**Figure 1:**
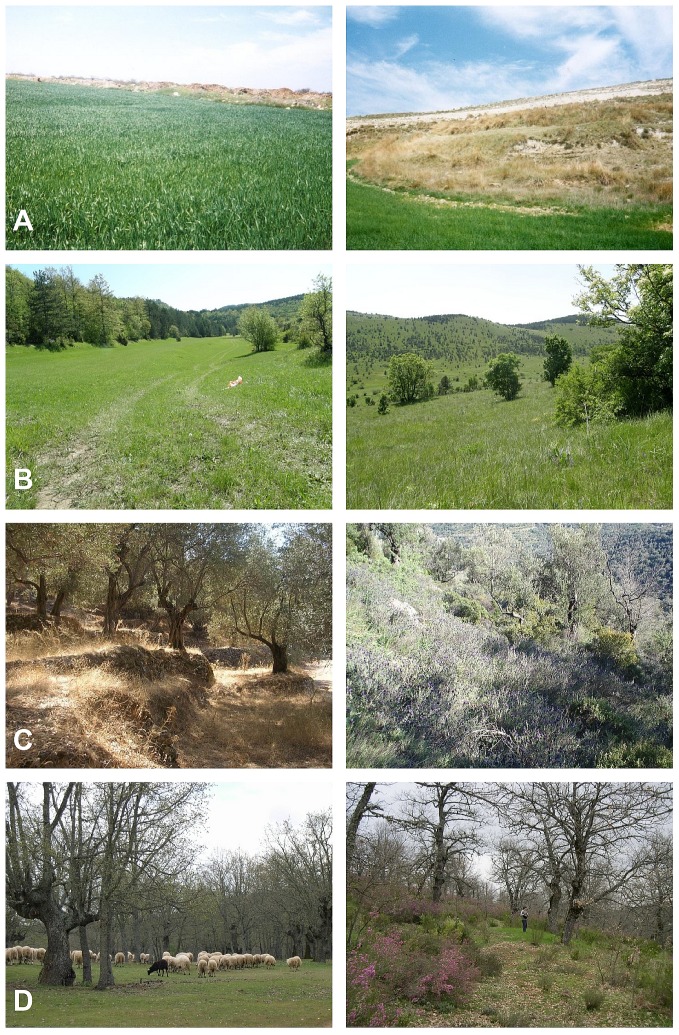
Examples of active and abandoned farmlands. (a) arable land in Burgos province, Spain; (b) grassland in a North Adriatic pastoral landscape, Croatia; (c) permanent crops: Olive groves on Lesvos, Greece; (d) agroforestry: *Quercus pyrenaica* dehesa in León province, Spain (sources: (a) J. Arroyo; (b) I. Kosić; (c) T. Plieninger, T. Kizos; (d) A. Taboada).

Our methodology was derived from the guidelines of the Collaboration for Environmental Evidence [37]; following these standards, a sampling protocol was peer-reviewed and published a priori (online repository: [23]). A Preferred Reporting Items for Systematic Reviews and Meta-Analyses (PRISMA) checklist was applied (Table S1). The minimum requirement for inclusion of a case study in the meta-analysis was that it reported summary data on plant or animal species richness or abundance comparing managed versus abandoned farmland. While species richness is argued to be a limited indicator of biodiversity, it is by far the most commonly used proxy for biodiversity in the primary studies that we evaluated. Among the simplest and most robust diversity measures available [38], [39], it underlies many ecological models and conservation strategies [40]. It is important to note that whether or not a given outcome, in terms of species richness or some other measure of biodiversity, is a “desirable” outcome is subjective, and will vary by region, landscape, and social factors and is beyond the scope of this study. The following definitions and study inclusion criteria were used:

- *Relevant populations*: Plant and animal populations that may change with the abandonment of agroforestry, arable land, pastures, and permanent crops (Figure 1). We based our definitions on the CORINE land cover nomenclature to delimit agroforestry, arable land, pastures, and permanent crops [41]. “Agroforestry” is defined as annual crops or pasture growing with forestry species such that the two interact; “arable land” refers to irrigated or non-irrigated lands used for annually cultivated and harvested crops; “pastures” are characterized by dense herbaceous cover, generally grazed or harvested for fodder; and “permanent crops” are crops that persist and are harvested over a longer than annual timeframe [41].
- *Relevant exposure*: The complete or partial abandonment of livestock grazing and/or crop cultivation. We understood abandonment as the ceasing of cultivation or grazing on farmland over a period of at least five years.
- *Types of comparators*: Comparisons between species richness and abundance before and after abandonment of particular sites and comparisons of abandoned land to adjacent reference farmland at the same moment in time (“space-for-time substitution” [42]).
- *Relevant outcomes*: Quantitative measures of richness and/or abundance of terrestrial plant and animal species. Only taxonomic group, not individual species abundances were included.
- *Relevant types of study design*: Observational field studies and manipulative field experiments. Control plots that were not abandoned should be in similar ecological settings, ideally close to abandoned plots.

We searched the following databases for relevant documents: ISI Web of Science, BIOSIS Citation Index, CAB Abstracts, Scopus, ProQuest Agricola, and ProQuest Dissertations & Theses. To minimize publication bias, we additionally included grey literature by considering the first 50 pdf and word documents provided by each of the following sources: Google, Google Scholar, and Dogpile. We considered studies in English, French, Italian, Portuguese, and Spanish. Search terms referred to the defined population, intervention and outcomes. Terms were broad enough to capture all relevant papers. The following logical search string was used: (abandon* OR “old fields” OR fallow) AND (biodiversity OR richness OR abundance OR composition OR assemblage) AND Mediterranean. Titles and abstracts were stored in a single Endnote database.

The search was performed in May 2013, yielding a total of 2012 studies. We obtained an additional 3 studies from colleagues. After removal of duplicates, 1240 studies remained. Study selection was a three-stage process. First, 632 studies with relevant titles were selected. Second, selection was made based on abstracts, after which 204 studies remained. To be considered in the meta-analysis, a study had to provide summary data (i.e. mean, standard deviation, and sample size) for species richness or abundance on managed vs. abandoned farmlands. When studies had collected relevant data but not published the required summary data, authors were contacted by e-mail. We contacted 33 authors, received information from 24 of them, and processed 19 of these datasets. Full paper content was assessed in the third stage, leaving 51 studies that provided all the information needed for the meta-analysis (means, standard deviations, sample sizes etc.) (Figure S1, see Table S2 for full references).

Repeatability of study inclusion was checked through a random subset of ca. 10% of references whose titles (150 papers) and abstracts (65 papers) were assessed by an independent reviewer. Inclusion consistency was calculated using kappa statistics [43]. Agreement between reviewers was good in both steps (k = 0.61 in the first stage and k = 0.72 in the second stage).

Study quality was assessed before data extraction. All articles that were finally selected met the requirements specified by our systematic review protocol.

### Data Treatment and Analysis

Observations of multiple taxa, different study sites, and/or different measurement times within one study were included separately in the dataset and considered independently. For each observation, we extracted means, standard deviations, and sample sizes (see Table S3). When summary statistics were not presented numerically, they were extracted from tables and graphs, using image analysis software in some cases. If original data were provided but summary statistics were lacking, summary statistics were calculated on the basis of raw data. In cases of insufficient information, corresponding authors were contacted to gather the required data. Location data given in the study were used to obtain parameter estimates for explanatory variables from other data sources (GoogleEarth, European Environment Agency, WorldClim) in order to extract variables that were not provided in the studies (Table 1, Table S4). The spatial resolution of WorldClim data is 1 km^2^. We considered a total of 8 independent variables (Table 1, plus 3 less important variables in Table S5).

**Table 1:** Explanatory variables provided by primary studies and additional data sources that were included in the meta-analysis and percentage of observations for which these data could be gathered.

Explanatory variable	Description	Source	Plant species richness (%)	Fungi species richness (%)	Animal species richness (%)	Animal abundance (%)
Unit size	Sample unit size (m^2^)	Primary studies	93	100	-	-
Extent	Extent of study area (km^2^)	Primary studies, GoogleEarth	95	100	100	100
Time since abandonment	Time elapsed since land was abandoned (years)	Primary studies	98	100	86	85
Previous land use	Agroforestry/arable land/pastures/permanent crops	Primary studies	100	100	100	100
Landform	Situated in mountain/lowland area	European Environment Agency	100	100	100	100
Protected area status	Situated in NATURA 2000 network of protected areas	European Environment Agency	88%	100	100	100
Temperature	Mean annual temperature (°C)	WorldClim	100	100	100	100
Precipitation	Mean annual precipitation (mm)	WorldClim	100	100	100	100

Data were synthesized through meta-analysis to address the overall effects of land abandonment on plant and animal richness and abundance. In meta-analysis, effect size is a difference value relative to the standard deviation. Additional aspects were addressed through meta-regression and sub-group analyses. Specifically, we measured the effect size of each case by the standardized mean difference, *d* = (*μ*_1_−*μ*_2_)/*sd_p_*. *μ*_1_ and *μ*_2_ are means of a focal dependent variable (e.g. population density and diversity) after and before land abandonment, respectively. The pooled standard deviation is *sd_p_* and equals the square root of ((*n*_1_−1)*sd*_1_^2^+(*n*_2_−1)*sd*_2_^2^)/(*n*_1_+*n*_2_−2), where *n*_1_ and *sd*_1_ are the sample size and standard deviation for experiments after land abandonment, and *n*_2_ and *sd*_2_ land without abandonment. The heterogeneity analysis of the data was examined using a Q-test, and the significance of the null hypothesis (i.e. the effect size equals zero) was examined by a Z-test as in [44]. We further conducted a meta-regression on temperature and precipitation as continuous moderators. All calculation was done by using the *metafor* package [45] in R software [46].

## Results

**Figure 2:**
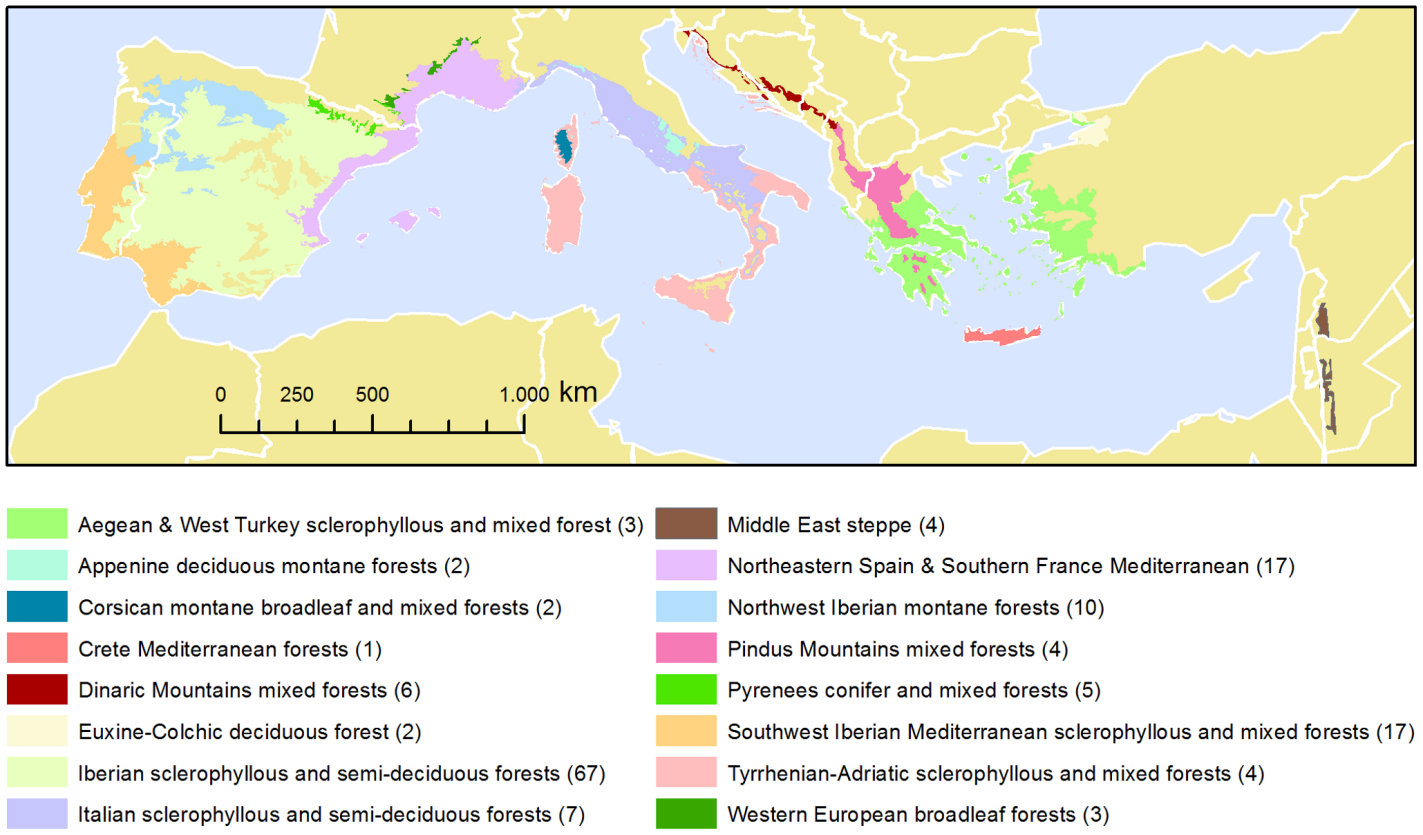
Map of ecological regions included in the analysis. Numbers in brackets specify the number of cases considered per ecological region.

Overall, our data set included 154 cases in 51 individual studies, published between 1974 and 2013 (Table S1). In particular, we found 89 cases in 38 primary studies for plant species richness (including fungi species richness), 24 cases in 14 studies for animal species richness, and 21 cases in 10 studies for animal abundance. Eighty-nine percent of cases of animal species richness and 76% of the cases of animal abundance referred to arthropods. Forty percent of cases considered agroforestry, 27% pastures, 20% permanent crops, and 12% arable land (Table 2). Iberian semi-sclerophyllous and semi-deciduous forests (44% of cases), Northeastern Spain and Southern France Mediterranean (11%), and Southwest Iberian Mediterranean sclerophyllous and mixed forests (11%) were among the most intensively studied ecological regions (Figure 2).

**Table 2:** Structure of the data set for comparing managed versus abandoned farmlands (number of cases).

Taxa	Agroforestry	Arable land	Pastures	Permanent crops
All	62	19	41	32
Fungi	4	0	0	0
Plants	20	4	33	28
Animals (richness)	28	8	5	3
…Arthropods (richness)	24	7	5	3
…Birds (richness)	4	1	0	0
Animals (abundance)	10	7	3	1
…Arthropods (abundance)	6	6	3	1
…Birds (abundance)	4	1	0	0

**Figure 3:**
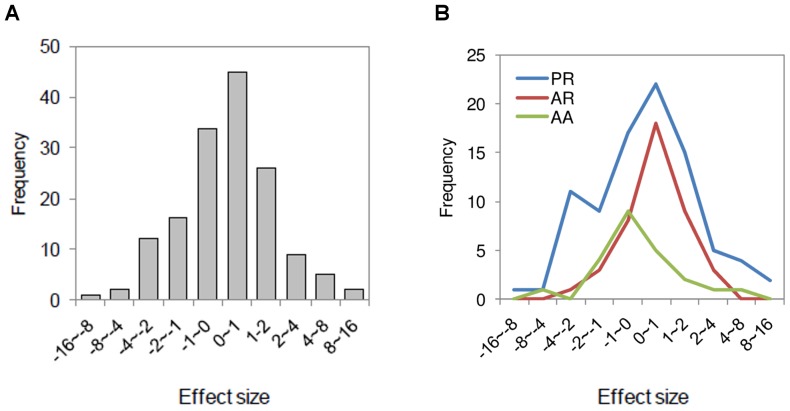
Frequency distribution of effect sizes for plant species richness, animal species richness and animal abundance (A) together and (B) separately. Mean difference effect size, g, and a mixed (random) effects model were used (PR – plant species richness; AR – animal species richness; AA – animal abundance).

Our meta-analysis (using a fixed effect model) revealed that – when analyzed together – plant and animal species richness and abundance values slightly increased after land abandonment (Effect Size Point Estimate ES = 0.2109), and this overall effect was significant (Z = 5.5991, P<0.0001). However, heterogeneity was high (Q = 1048.89, P<0.0001) as outcomes were divided. In 91 (59%) of the 154 cases, species richness and abundance values were higher on abandoned land compared to managed farmland; in the remaining 63 cases (41%), values were lower. Fifty-one cases (33%) had positive effect sizes, indicating a significant increase of species richness or abundance after abandonment. Forty-four cases (29%) had negative effect sizes (indicating significant decreases of species richness or abundance), while in 59 cases (38%) effect sizes did not differ significantly from zero (i.e., SD included 0) (Table S3). Following Cohen's classification [47], 54% of the cases had large effect sizes (>0.8), 36% had medium effect sizes (0.2–0.8), and 8% had small (<0.2) effect sizes (Figure 3). Four cases were not replicated. Due to the heterogeneous effect size, mixed effect models were used to examine the different factors.

**Figure 4:**
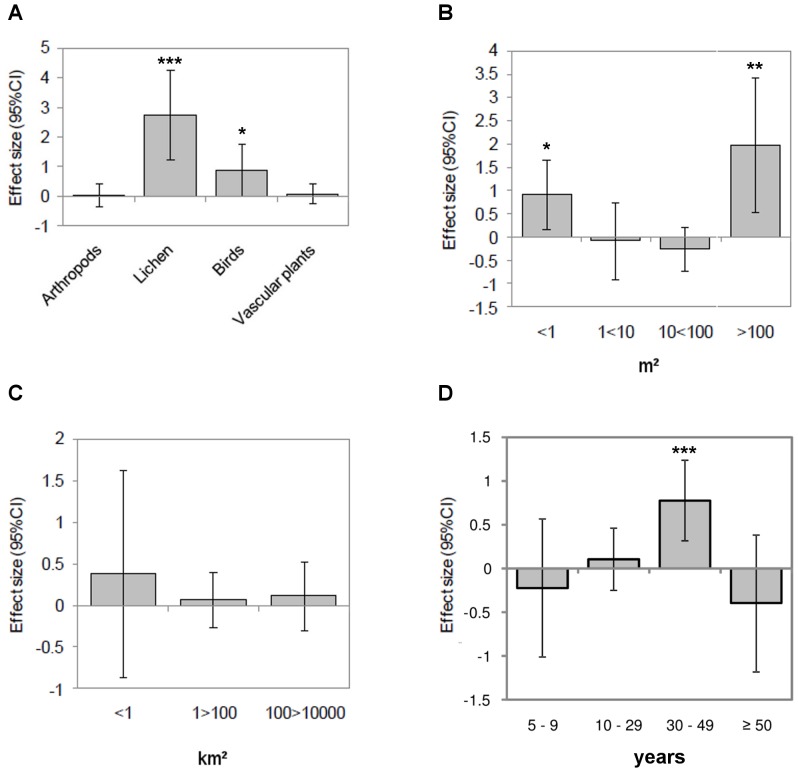
Effect size (95% CI) of land abandonment for (A) taxon; (B) unit size of study; (C) extent of study area; (D) time since abandonment. Q-test shows significant different effect sizes between groups (heterogeneity) for taxon (Q = 16.95, P = 0.002) and time since abandonment (Q = 12.68, P = 0.013), but not for extent (Q = 0.86, P = 0.8356).

Differences in species richness were most pronounced in the fungi (Table 3). However, land abandonment also showed significant increases in animal and plant species richness. Among the taxa, we found significantly higher effect sizes for lichen and birds on abandoned land, while there was no global effect of land abandonment on arthropod and vascular plants (Table 3, Figure 4A). Thirty-four cases (38%) had a positive effect size for plant species richness, while 28 cases (31%) had a negative effect size and 27 cases (30%) were not significantly different from zero. Among studies of animal species richness, 14 cases (32%) had positive and 7 cases (16%) negative effect sizes, and in 23 cases (52%) effect sizes were not significantly different from zero. Animal abundance studies had positive effect sizes in 3 cases (14%), negative effect sizes in 9 cases (43%) and insignificant deviations from zero in 9 cases (43%).

**Table 3:** Summary of the meta-analysis.

Moderator (Q, P)	ES	SE	Z	P	95%CI Lower	95%CI Upper	N
*Kingdom (95.36, <0.0001)*							
Animals	0.1625	0.0628	2.5876	0.0097	0.0394	0.2856	65
Fungi	1.2717	0.1384	9.1895	<0.0001	1.0004	1.5429	4
Plants	0.1028	0.0501	2.0544	0.0399	0.0047	0.2010	85
*Taxon (16.95, 0.002)*							
Arthropods	0.0424	0.2026	0.2091	0.8344	−0.3548	0.4395	55
Birds	0.8829	0.4445	1.9862	0.0470	0.0117	1.7541	10
Lichen	2.7541	0.7738	3.5594	0.0004	1.2375	4.2706	4
Vascular plants	0.0909	0.1673	0.5432	0.5870	−0.2371	0.4189	85
*Unit size(14.02, 0.0072)*							
<1 m^2^	0.9154	0.3793	2.4133	0.0158	0.1719	1.6589	18
1<10 m^2^	−0.0827	0.4236	−0.1953	0.8452	−0.9130	0.7475	13
10<100 m^2^	−0.2353	0.2421	−0.9722	0.3310	−0.7097	0.2391	47
>100 m^2^	1.9763	0.7357	2.6862	0.0072	0.5343	3.4182	5
*Extent (0.86, 0.8356)*							
<1 km^2^	0.3797	0.6310	0.6018	0.5473	−0.8570	1.6164	11
1>100 km^2^	0.0713	0.1673	0.4263	0.6699	−0.2566	0.3992	91
100>10000 km^2^	0.1196	0.2135	0.5601	0.5754	−0.2989	0.5381	48
*Time since abandonment (12.68, 0.013)*							
5–9 years	−0.2189	0.3996	−0.5477	0.5839	−1.0021	0.5644	16
10–29 years	0.1061	0.1807	0.5869	0.5572	−0.2481	0.4602	72
30–49 years	0.7822	0.2353	3.3244	0.0009	0.3210	1.2433	42
≥50 years	−0.3925	0.3963	−0.9903	0.3220	−1.1693	0.3843	13
*Previous land use (18.72, 0.0009)*							
Agroforestry	0.5765	0.1880	3.0666	0.0022	0.2080	0.9449	62
Arable land	0.7180	0.3559	2.0173	0.0437	0.0204	1.4155	19
Pastures	−0.5072	0.2254	−2.2500	0.0244	−0.9491	−0.0654	41
Permanent crops	0.1256	0.2938	0.4274	0.6691	−0.4502	0.7013	32
*Landform (9.76, 0.0076)*							
Mountain area	−0.0028	0.1471	−0.0191	0.9847	−0.2911	0.2854	107
Lowland area	0.7579	0.2426	3.1245	0.0018	0.2825	1.2333	47
*Protected area status (0.31, 0.8553)*							
Inside NATURA 2000 area	0.0430	0.1511	0.2843	0.7762	−0.2532	0.3392	107
Outside NATURA 2000 area	0.1242	0.2580	0.4814	0.6302	−0.3814	0.6298	37
*Climate (12.18,0.0023)*							
Intercept	2.1029	1.0552	1.9929	0.0463	0.0347	4.1711	150
Temperature	−0.0425	0.0602	−0.7054	0.4806	−0.1606	0.0756	150
Precipitation	−0.0021	0.0006	−3.2903	0.0010	−0.0034	−0.0009	150

Q  =  Q-test for heterogeneity (including P value); ES  =  effect size point estimate; SE  =  standard error; Z  =  two-tail Z-test; 95%CI Lower  =  lower confidence interval (95%); 95%CI Upper  =  upper confidence interval (95%); N  =  Number of records of each category. Categorical moderators are analyzed using mixed effect models; the last moderator “Climate” of two continuous variables is assessed by a meta-analytic model. The cursive fonts indicate the specific variables and their Q and P values.

As for spatial-temporal patterns, effect size of land abandonment (assessed for plant species richness only) was positive for small (<1 m^2^) and large (>100 m^2^) unit sizes, but insignificant for medium-sized units (1<10 m^2^; 10<100 m^2^) (Table 3, Figure 4B). No clear patterns emerged for the extent of the study areas (Figure 4C). The number of years since abandonment did have a significant impact on effect size; but only studies that covered an abandonment period of thirty to forty-nine years (not those with fifty or more years or less than thirty years of abandonment) showed significant increases in species richness and abundance (Table 3, Figure 4D).

**Figure 5:**
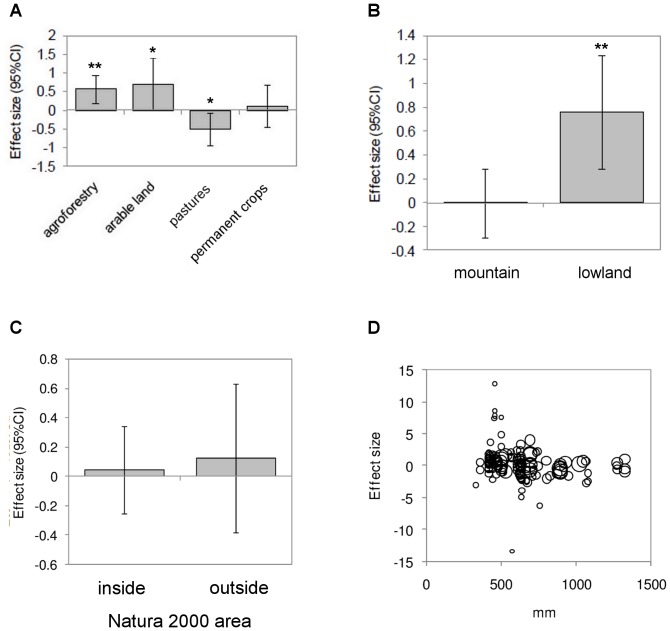
Effect size (95% CI) of land abandonment for (A) previous land use; (B) landform; (C) protected areas; (D) precipitation. Q-test shows significant different effect sizes (heterogeneity) between groups (A: Q = 18.72, P = 0.009 and B: Q = 9.76, P = 0.0076), but not for C: (Q = 0.31, P = 0.8553). D displays a bubble plot of the relationship between effect size and precipitation, with the size of the bubbles scaled according to the reciprocal of the standard deviation of the effect size.

Agroforestry, arable land, and pastures showed significantly different effect sizes between groups (Table 3, Figure 5A). On agroforestry and arable land, species richness and abundance increased after abandonment, while it decreased on pastures. Permanent crops did not exhibit significant effects.

Effect sizes of studies performed in mountains and lowlands significantly differed from each other (Table 3, Figure 5B), with abandonment in lowland areas showing stronger increases in plant and animal species richness and abundance. No differences were found comparing land inside and outside of the European network of protected areas (NATURA 2000, Table 3, Figure 5C). Temperature did not show significant effects, whereas areas with high precipitation showed significant declines of plant and animal species richness and abundance after abandonment (Table 3, Figure 5D).

## Discussion

Land abandonment potentially has substantial environmental and socio-economic consequences [5]. This study presents the first formal meta-analysis that examines the particular impacts of land abandonment on biodiversity, using animal species richness, animal abundance, and plant species richness as indicators. The analysis focused on the Mediterranean Basin, an area of comparable climate where land abandonment is prevalent. The meta-analysis revealed that land abandonment has been shown to slightly but significantly result in increases in plant and animal species richness and abundance. However, heterogeneity in responses to abandonment was high. Among the 154 empirical cases used in the meta-analysis, many pointed to increases, and others to decreases, in biodiversity after farmland abandonment. For example, when a simply structured vineyard in Israel was abandoned, the mean species richness values of perennial plants (between vine rows) increased from 0.0 to 2.3 species per m^2^ [48]. In contrast, mean plant species richness declined after abandonment from 16.4 to 12.4 species per m^2^ in a multifunctional grazing system in Northern Spain [49], or from 38.4 to 21.8 species per 100 m^2^ in a traditionally cultivated chestnut grove in Southern France [50]. In some empirical studies, effect sizes went in different directions when different species groups [51] or different farmland habitats [52] were investigated. Using a diversity of indicators, a qualitative, global review of land abandonment came to similar insight, with 77 studies pointing to biodiversity losses, but another 39 studies reporting increasing biodiversity [3]. Thus, the responses of species richness and abundance are not consistent enough to support general conclusions about biodiversity trends on abandoned lands in the Mediterranean. Rather, these responses seem strongly mediated by the specifics of each case study, whether they pertain to spatial-temporal scale, land-use, landforms, climate, or other parameters.

### Variation in Land Abandonment Impacts

In regard to objective 1), the diverging views on increases or decreases in plant and animal populations that result from land abandonment can be partly explained by the different taxonomic groups involved. All three kingdoms (animals, fungi, plants) showed an overall positive effect size after abandonment, but the strongest one was found for lichen (remembering that all lichen cases were taken from one publication only). Bird species richness also showed clear increases in response to land abandonment. The finding that lichen and birds are more sensitive to land abandonment than other taxa may explain why the effect size for small (<1 m^2^) and large study units (>100 m^2^) was significant, as studies on lichen are conducted at finer scales while studies on birds are mainly carried out at broader scales. Responses of vascular plant richness were heterogeneous, with some plant communities favored by agricultural management (very likely those composed of ruderal, stress tolerant, and competitive farmland species) and some (very likely those composed of shrubland and woodland species) favored by abandonment. A meta-analysis of land abandonment effects on bird distribution changes also found such heterogeneous differences, with decreasing occurrence of farmland bird species and increasing occurrence of woodland and shrubland species after abandonment [19].

As for objective 2), our results showed that the temporal dimension of land abandonment studies is important [53]. Plant species richness often increases and exhibits strong dynamics in the first years after abandonment, but later species composition becomes more stable and species richness decreases. The intermediate disturbance hypothesis offers one potential explanation, as it predicts that plant competition has a greater influence on plant community development when it is not interrupted by disturbances such as cultivation, drought or grazing [50], [54]. In a highly competitive environment, less successful competitors are often eventually suppressed. In our meta-analysis however, only studies considering an abandonment period of 30–49 years showed significant increases in species richness. Obviously, several decades are needed until colonizers in the regional species pool trigger community succession. Therefore, our results highlight that comparatively long time periods are required before general increases in species richness and abundance can be detected. However, a (non-significant) decline in species richness after an abandonment period of 50 or more years may indicate that exclusion processes eventually follow colonization processes in many of the case studies.

Substantially different outcomes were revealed for different agricultural systems [55] when objective 3) was investigated, confirming previous studies of the influence of farm-level attributes on biodiversity [56]. Species richness and abundance generally increased on cultivated habitats (arable land, agroforestry) after abandonment, and decreased in abandoned pastures. Effect sizes in Figure 5B suggest an order from increased to decreased species richness, from agroforestry (+), to arable land (+), to permanent crops (non-significant), and to pasture (-). This may be related to a gradient in plant height and in “naturalness” of the vegetation types. Cultivated habitats are generally more disturbed by agricultural activities and more distant in species composition from natural ecosystems than are pastures. Accordingly, stronger increases in species richness following land abandonment could be expected for cultivated lands as reduced soil disturbance allows longer-lived plants to become part of and “de-simplify” the available habitats [57]. However, mechanisms of species increase or decrease in these habitats are controlled by plot-level variation in landscape structure (in particular, the amount of semi-natural habitats), land-use legacies, and/or management intensity [58]. Effects on biodiversity values are also likely to vary within arable or pasture lands for the same reason. Biodiversity impacts after abandonment may differ between highly mechanized and simplified croplands for example, and traditionally grazed native pasture land where grazing may moderate competitive exclusion [59]. The “nature value” of farmlands prior to abandonment would be worth exploring as variables in the analyses, but spatially explicit information on the occurrence of high-nature value farmland is not available at the European level at present and therefore hard to consider in a meta-analysis. Surprisingly, we did not find differences between studies carried out inside and outside the network of protected areas that covers around 18% of the European Union.

Regarding objective 4), some effects of landforms, climate, and other contextual factors were revealed. Particularly influential was the ecological region of the Mediterranean Basin where the study was carried out. Land abandonment impacts were more negative to species richness in areas of higher precipitation (Table 3), i.e. in those environments of the Mediterranean where climatic conditions are less challenging. This pattern supports prevailing notions about non-equilibrium systems [60]; in accordance with non-equilibrium concepts, in areas where abiotic factors do not limit competition as a major driver, human disturbance may favor greater species richness. Therefore, higher precipitation may lead to high levels of competitive exclusion when disturbance from agriculture ceases.

### Limitations of the Study

When interpreting the results of our meta-analysis, several caveats need to be taken into account. Although meta-analysis is acknowledged as a straightforward method that yields robust quantitative results, relevant information reported in the empirical studies used may be lost, and some relevant studies may be missed in the selection process. Our search found that many papers could not be included because necessary summary statistics were not provided. This information could be gained from some, but by no means all, authors. We tried to ensure comparability between primary studies by restricting our analysis to the Mediterranean Basin, and by adding standardized information (temperature, precipitation, landform, protected area status) from databases that covered the whole Basin (or at least the European part of it). However, the strong variability that we found indicates that the relationship between land use and biodiversity in the Mediterranean may be too complex for general conclusions.

Our analysis may be further limited because of publication bias, the idea that studies reporting significant differences are more frequently published than studies that do not find significant differences. We tried to minimize publication bias by not only including studies that were published in high impact journals but also results from theses, national-level periodicals, internet documents and other forms of “grey literature”. The distribution of effect sizes of our 154 cases is rather symmetric and normal (Figure 3), so it does not indicate any obvious publication bias. Rather, most effect sizes are moderate, and only few are large. Before-after studies may be sensitive to random factors such good or bad years in terms of rainfall. However, only 2 out of the 154 cases were before-after studies, so we believe that the influence of such factors on the outcome of our meta-analysis is low.

Another issue to be discussed is whether the inclusion of several cases per published study leads to pseudoreplication. Having the number of cases exceed the number of studies is very common in meta-analysis studies [61]. It can be addressed by randomly selecting one case per study and examining whether the confidence interval of the effect size for these selected cases is different from the confidence interval for all cases. This is statistically equivalent to comparing the confidence intervals of the mean effect size for each study, calculated from a mixed-effect model using “study” as a factor, with the confidence interval of the effect size for all cases (from Figure 3A). A lack of overlap in 83% confidence intervals represents a significant difference at P = 0.05 (note that a lack of overlap in 95% confidence intervals indicates a statistically significant difference at much smaller thresholds of P value [62]). The 83% confidence interval in Fig. 3A is −2.115, 2.217, and the 83% confidence interval for the “ES section of ‘study’” in Table S5 is −2.876, 1.497. Consequently, the effect of pseudoreplication is not significant and our way of analysis is acceptable.

Perhaps the most important limitation to address is the selection of biodiversity indicators. As have many other meta-analyses [57], [63], [64], we focused our study on species richness. However, species richness can be an unreliable indicator of biodiversity [53], and more sophisticated comparisons based on species composition would be more informative [65]. In addition to species richness, we considered species abundance for the assessment of the biodiversity impacts of land abandonment, as diminishing abundance may translate into reduced genetic diversity of populations [66]. Our approach did not allow us to consider studies of other dimensions of biodiversity, for example of differences in ecosystem diversity [67], or of population changes in individual species [19]. If these parameters were to be included, we suspect the overall impacts of land abandonment in Europe might more often lead to decreases in biodiversity [3], [18]. In addition, our meta-analysis did not assess species composition. As a result losses in farmland biodiversity and especially agrobiodiversity that accompany many abandonment processes [27] might have been overlooked. Similarly, conceptions of what constitutes “land abandonment” can vary substantially [68].

### Research Needs

Our sample of primary studies was not distributed evenly across the Mediterranean Basin. Most cases, 96%, were from Europe, with a single country – Spain – contributing 56% of all cases. In contrast, not a single one was assessed from the southern shore of the Mediterranean Basin. This might reflect a biased selection of study cases, but in large part can also be attributed to the fact that land abandonment is a particular phenomenon of the European Mediterranean, as land use pressure remains high in African and Asian regions [25], [69]. Given that they represent regional-specific land-use systems, the *dehesa* and *montado* agroforestry systems of the Iberian Peninsula received a lot of attention in studies. In contrast, the biodiversity outcomes of land abandonment were comparatively little studied for arable land. Also, not all taxa received equal attention, and current studies do not allow identifying the specific kinds of plant and animal communities that are favored or hampered by land abandonment.

Future research studying land abandonment should strive to fill the gaps identified in this paper by focusing on neglected taxa and regions and by studying effects on species composition, turnover, and functional biodiversity rather than species numbers. Other promising directions might be closer examination of biodiversity outcomes under different intensities of land management (e.g., intensive crop cultivation versus high nature value farming versus organic agriculture) and land abandonment (e.g., complete versus “mild” abandonment of traditional olive cultivation), and under different land tenure regimes [70]. In particular, the landscape context of land abandonment and biodiversity needs much more attention.

## Conclusions

It is challenging to explain the contrasting impacts of a complex and spatially diverse process such as land abandonment [18]. Synthesizing the results of 154 cases throughout the Mediterranean Basin, this meta-analysis indicates a slight increase in overall species richness and abundance after land abandonment. The effects of land abandonment on biodiversity were mediated by a broad set of drivers. As a result, the directions and intensities of response in species richness and abundance to land abandonment were heterogeneous across the Basin.

Our results point out that neither “rewilding” nor “high nature value farming” alone offer “one-size-fits-all” policy solutions for addressing biodiversity conservation following land abandonment in the Mediterranean Basin. Rather, agri-environmental and other approaches need to be tailored to the local ecosystem, landscape, and land-use context. For example, our study gives hints that abandonment of some plots within simply structured cultivated landscapes may increase landscape heterogeneity and habitat diversity and thus contribute to ecological restoration [71], [72]. In contrast, land abandonment in pastoral landscapes may be detrimental, in particular to farmland biodiversity that is linked to active human intervention [14]. In fact, land abandonment affects extensively managed Mediterranean farmland much more than intensively managed farmland [73], so that the scenario of decreasing farmland biodiversity seems to be the more common case. An ideal configuration may be a landscape mosaic with patches of differing successional stages and management types, with each stage and type benefitting particular taxonomic groups [3]. An important task for the future will be to develop a typology of potential responses to land abandonment.

Given prevailing socio-economic conditions, land abandonment will continue in many parts of the Mediterranean Basin. An outcome of larger agricultural change, the process is unlikely to be efficiently addressed by broad agricultural policy and even less by biodiversity conservation programs [35]. Our results call, firstly, for spatially explicit targeting of policies toward specific hotspots of land abandonment [5], [26]. Secondly, policies should address only those farmlands where otherwise uncontrolled abandonment would lead to socially undesired outcomes for biodiversity and ecosystem services. The results of this study point out that abandonment of pasture lands may need particular targeting by agri-environmental policies to maintain or restore biodiversity values. The strong role of factors at the farm and landscape scales that was revealed by the analysis indicates that purposeful management at these scales can have a powerful impact on biodiversity, for example in situations where ecological processes such as dispersal and regeneration are disrupted by surrounding industrial agriculture [7]. A context-specific approach requires assessments of broad sets of biodiversity and ecosystem services at the landscape scale as well as cross-sectoral rural development approaches.

## Supporting Information

Figure S1 Flow diagram reporting the number of records identified, excluded, and added during the screening process.
(PDF)

Table S1 PRISMA (Preferred Reporting Items for Systematic Reviews and Meta-Analyses) checklist.
(PDF)

Table S2 Full references for the 51 studies included in the meta-analysis.
(PDF)

Table S3 Cases included in the meta-analysis: Dependent variables.
(PDF)

Table S4 Cases included in the meta-analysis: Independent variables.
(PDF)

Table S5 Summary of additional variables included in the meta-analysis.
(PDF)
