# Supplementary material for: The Impact of Land Abandonment on Species Richness and Abundance in the Mediterranean Basin: A Meta-Analysis
Source: PLoS One. 2014 May 27;9(5):e98355. doi: 10.1371/journal.pone.0098355 (PMC4035294; doi:10.1371/journal.pone.0098355)

Figure S1. Flow diagram reporting the number of records identified, excluded and added during the screening process.

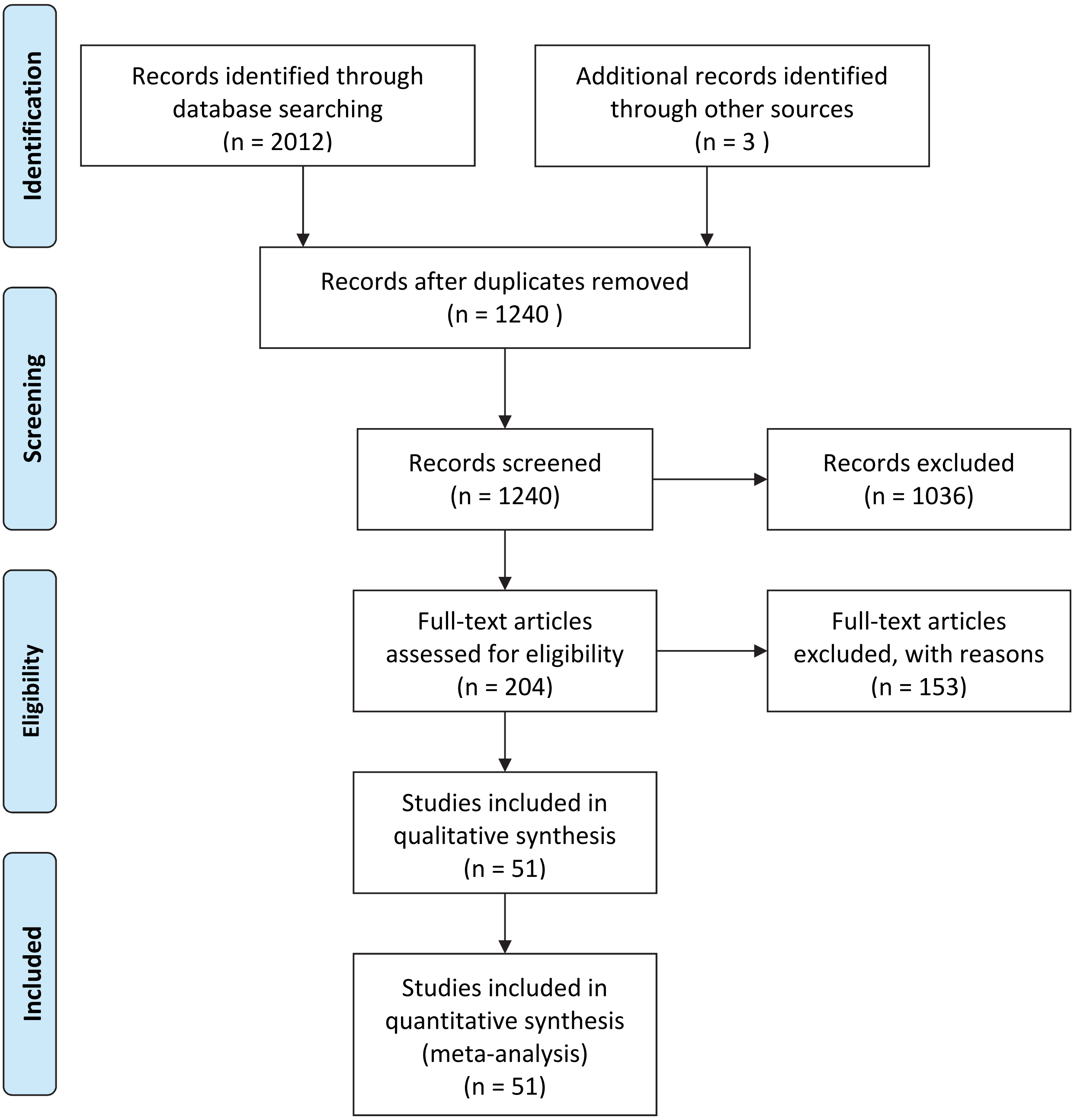

Supplement: Figure S1 — Flow diagram reporting the number of records identified, excluded, and added during the screening process. (PDF) [file pone.0098355.s001.pdf]
