# Supplementary material for: The Impact of Land Abandonment on Species Richness and Abundance in the Mediterranean Basin: A Meta-Analysis
Source: PLoS One. 2014 May 27;9(5):e98355. doi: 10.1371/journal.pone.0098355 (PMC4035294; doi:10.1371/journal.pone.0098355)
Supplement: Table S2 — Full references for the 51 studies included in the meta-analysis. (PDF) [file pone.0098355.s003.pdf]

Table S2. Full references of 51 studies included in the meta-analysis.

Allen, H. D., R. E. Randall, G. S. Amable, and B. J. Devereux. 2006. The impact of changing olive cultivation practices on the ground flora of olive groves in the Messara and. Psiloritis regions, Crete, Greece. Land Degradation & Development 17:249-273.

Andres, C. and F. Ojeda. 2002. Effects of afforestation with pines on woody plant diversity of Mediterranean heathlands in southern Spain. Biodiversity and Conservation 11:1511-1520.

Aragon, G., R. Lopez, and I. Martinez. 2010. Effects of Mediterranean dehesa management on epiphytic lichens. Science of the Total Environment 409:116-122.

Arroyo, J., J. C. Iturrondobeitia, C. Rad, and S. González-Carcedo. 2005. Oribatid mite (Acari) community structure in steppic habitats of Burgos Province, central northern Spain. Journal of Natural History 39:3453-3470.

Azcarate, F. M. and B. Peco. 2012. Abandonment of grazing in a Mediterranean grassland area: consequences for ant assemblages. Insect Conservation and Diversity 5:279-288.

Barriga, J. C., L. Lassaletta, and A. G. Moreno. 2010. Ground-living spider assemblages from Mediterranean habitats under different management conditions. Journal of Arachnology 38:258-269.

Bonanomi, G., S. Caporaso, and M. Allegrezza. 2009. Effects of nitrogen enrichment, plant litter removal and cutting on a species-rich Mediterranean calcareous grassland. Plant Biosystems 143:443-455.

Bonet, A. 2004. Secondary succession of semi-arid Mediterranean old-fields in south-eastern Spain: insights for conservation and restoration of degraded lands. Journal of Arid Environments 56:213-233.

Borghesio, L., C. Palestrini, and E. Balletto. 2005. Butterfly ecology and conservation of a site in the pre-apennines of piedmont (NW Italy). Revue d'Ecologie (La Terre et la Vie) 60:33-44.

Carmona, C. P., F. M. Azcarate, F. de Bello, H. S. Ollero, J. Leps, and B. Peco. 2012. Taxonomical and functional diversity turnover in Mediterranean grasslands: interactions between grazing, habitat type and rainfall. Journal of Applied Ecology 49:1084-1093.

Castro, H., V. Lehsten, S. Lavorel, and H. Freitas. 2010. Functional response traits in relation to land use change in the Montado. Agriculture Ecosystems & Environment 137:183-191.

Catorci, A., S. Cesaretti, R. Gatti, and G. Ottaviani. 2011a. Abiotic and biotic changes due to spread of Brachypodium genuense (DC.) Roem. & Schult. in sub-Mediterranean meadows. Community Ecology 12:117-125.

Catorci, A., G. Ottaviani, and S. Cesaretti. 2011b. Functional and coenological changes under different long-term management conditions in Apennine meadows (central Italy). Phytocoenologia 41:45-58.

Celik, I., K. T. Yilmaz, H. Eswaran, A. Mermut, M. Dingil, Z. Kaya, A. Demirbas, I. Aksit, I. Ortas, M. Gok, C. Akpinar, T. Nagano, N. Ae, Y. K. Koca, and S. Kapur. 2011. Reconstructing the Past by Regenerating Biodiversity: A Treatise on Weed Contribution to Soil Quality at a Post-cultivation Succession. Pages 363-378 in S. Kapur, H. Eswaran, and W. E. H. Blum, editors. Sustainable Land Management: Learning from the Past for the Future. Springer-Verlag Berlin, Berlin.

Curt, T., B. Prevosto, M. Klesczewski, and J. Lepart. 2003. Post-grazing Scots pine colonization of mid-elevation heathlands: population structure, impact on vegetation composition and diversity. Annals of Forest Science 60:711-724.

David, J. F., S. Devernay, G. Loucougaray, and E. L. Floc'h. 1999. Belowground biodiversity in a Mediterranean landscape: relationships between saprophagous macroarthropod communities and vegetation structure. Biodiversity & Conservation 8:753-767.

de Bello, F. d., J. Leps, and M. T. Sebastia. 2006. Variations in species and functional plant diversity along climatic and grazing gradients. Ecography 29:801-810.

Debussche, M., J. Escarre, J. Lepart, C. Houssard, and S. Lavorel. 1996. Changes in Mediterranean plant succession: Old-fields revisited. Journal of Vegetation Science 7:519-526.

Fadda, S., F. Henry, J. Orgeas, P. Ponel, E. Buisson, and T. Dutoit. 2008. Consequences of the cessation of 3000 years of grazing on dry Mediterranean grassland ground-active beetle assemblages. Comptes Rendus Biologies 331:532-546.

Farris, E., R. Filigheddu, P. Deiana, G. A. Farris, and G. Garau. 2010. Short-term effects on sheep pastureland due to grazing abandonment in a Western Mediterranean island ecosystem: A multidisciplinary approach. Journal for Nature Conservation 18:258-267.

García-Tejero, S., S. Taboada, R. Tárrega, and J. M. Salgado. 2013. Land use changes and ground dwelling beetle conservation in extensive grazing dehesa systems of north-west Spain. Biological Conservation 161:58-66.

Gomez, C., D. Casellas, J. Oliveras, and J. M. Bas. 2003. Structure of ground-foraging ant assemblages in relation to land-use change in the northwestern Mediterranean region. Biodiversity and Conservation 12:2135-2146.

Gondard, H., F. Romane, M. Grandjanny, J. Q. Li, and J. Aronson. 2001. Plant species diversity changes in abandoned chestnut (Castanea sativa) groves in southern France. Biodiversity and Conservation 10:189-207.

Gondard, H., F. Romane, I. Santa-Regina, and S. Leonardi. 2006. Forest management and plant species diversity in chestnut stands of three Mediterranean areas. Biodiversity and Conservation 15:1129-1142.

Kosic, I. V., F. M. Tardella, and A. Catorci. 2012. Effect of management modification on the coenological composition of the North Adriatic pastoral landscape (Cicarija, Croatia). Hacquetia 11:17-46.

La Mantia, T., J. Ruhl, S. Pasta, D. G. Campisi, and G. Terrazzino. 2008. Structural analysis of woody species in Mediterranean old fields. Plant Biosystems 142:462-471.

Lesschen, J. P., L. H. Cammeraat, A. M. Kooijman, and B. van Wesemael. 2008. Development of spatial heterogeneity in vegetation and soil properties after land abandonment in a semi-arid ecosystem. Journal of Arid Environments 72:2082-2092.

Lopez-i-Gelats, F. and J. Bartolome. 2008. The effects of different kinds of livestock farming and abandonment on botanical diversity in mountain hay meadows. Pages 138-140 in A. Hopkins, T. Gustafsson, J. Bertilsson, G. Dalin, N. Nilsdotter-Linde, and E. Sporndly, editors. Biodiversity and animal feed: future challenges for grassland production. Proceedings of the 22nd General Meeting of the European Grassland Federation, Uppsala, Sweden, 9-12 June 2008.

Martinez-Duro, E., A. L. Luzuriaga, P. Ferrandis, A. Escudero, and J. M. Herranz. 2012. Does aboveground vegetation composition resemble soil seed bank during succession in specialized vegetation on gypsum soil? Ecological Research 27:43-51.

Mesléard, F., J. Lepart, P. Grillas, and A. Mauchamp. 1999. Effects of seasonal flooding and grazing on the vegetation of former ricefields in the Rhone delta (Southern France). Plant Ecology 145:101-114.

Ne'eman, G. and I. Izhaki. 1996. Colonization in an abandoned East-Mediterranean vineyard. Journal of Vegetation Science 7:465-472.

Pala, S. and C. Siniscalco. 2000. Vegetation dynamics in secondary prairies of a low altitude area in Val Borbera (Appennino Ligure-Piemontese, Province of Alessandra). Archivio Geobotanico 6:99-111.

Peco, B., C. P. Carmona, I. de Pablos, and F. M. Azcarate. 2012. Effects of grazing abandonment on functional and taxonomic diversity of Mediterranean grasslands. Agriculture Ecosystems & Environment 152:27-32.

Peco, B., A. M. Sanchez, and F. M. Azcarate. 2006. Abandonment in grazing systems: Consequences for vegetation and soil. Agriculture Ecosystems & Environment 113:284-294.

Plexida, S., A. Sfougaris, and N. Papadopoulos. 2012. Quantifying beetle and bird diversity in a Mediterranean mountain agro-ecosystem. Israel Journal of Ecology and Evolution 58:1-25.

Porto, M., O. Correia, and P. Beja. 2011. Long-term consequences of mechanical fuel management for the conservation of Mediterranean forest herb communities. Biodiversity and Conservation 20:2669-2691.

Potts, S. G., T. Petanidou, S. Roberts, C. O'Toole, A. Hulbert, and P. Willmer. 2006. Plant-pollinator biodiversity and pollination services in a complex Mediterranean landscape. Biological Conservation 129:519-529.

Pretto, F., L. Celesti-Grapow, E. Carli, and C. Blasi. 2010. Influence of past land use and current human disturbance on non-native plant species on small Italian islands. Plant Ecology 210:225-239.

Puerto, A. and M. Rico. 1988. Influence of tree canopy (Quercus rotundifolia Lam. and Quercus pyrenaica Willd.) on old field succession in marginal areas of central-western Spain. Acta Oecologica, Oecologia Plantarum 9:337-358.

Redondo P, B., E. Luis C, A. Puerto M, and J. M. Gomez. 1974. Descripcion de cuatro etapas de la sucesion secundaria en pastizales sayagueses. Revista Pastos 4:235-245.

Romane, F. and L. Valérino. 1997. Changements du paysage et biodiversité dans les châtaigneraies cévenoles (sud de la France). Ecologia Mediterranea 23:121-129.

Said, S., J. Gamisans, A. Bedecarrats, P. Delcros, and J. C. Rameau. 2001. Secondary succession in the Zicavo region of Corsica (Mediterranean island Climate effect. Annals of Forest Science 58:615-624.

Santana, J., M. Porto, L. Gordinho, L. Reino, and P. Beja. 2012. Long-term responses of Mediterranean birds to forest fuel management. Journal of Applied Ecology 49:632-643.

Santana, J., M. Porto, L. Reino, and P. Beja. 2011. Long-term understory recovery after mechanical fuel reduction in Mediterranean cork oak forests. Forest Ecology and Management 261:447-459.

Scalercio, S., N. Iannotta, and P. Brandmayr. 2007. The role of semi-natural and abandoned vegetation patches in sustaining lepidopteran diversity in an organic olive orchard. Bulletin of Insectology 60:13-22.

Schmitz, M. F., I. A. Sanchez, and I. de Aranzabal. 2007. Influence of management regimes of adjacent land uses on the woody plant richness of hedgerows in Spanish cultural landscapes. Biological Conservation 135:542-554.

Skornik, S., M. Vidrih, and M. Kaligaric. 2010. The effect of grazing pressure on species richness, composition and productivity in North Adriatic Karst pastures. Plant Biosystems 144:355-364.

Tarrega, R., L. Calvo, A. Taboada, S. Garcia-Tejero, and E. Marcos. 2009. Abandonment and management in Spanish dehesa systems: Effects on soil features and plant species richness and composition. Forest Ecology and Management 257:731-738.

Tatoni, T., F. Magnin, G. Bonin, and J. Vaudour. 1994. Secondary successions on abandoned cultivation terraces in calcareous Provence. I- Vegetation and soil. Acta Oecologica 15:431-447.

Verdasca, M. J., A. S. Leitao, J. Santana, M. Porto, S. Dias, and P. Beja. 2012. Forest fuel management as a conservation tool for early successional species under agricultural abandonment: The case of Mediterranean butterflies. Biological Conservation 146:14-23.

Zamora, J., J. R. Verdú, and E. Galante. 2007. Species richness in Mediterranean agroecosystems: Spatial and temporal analysis for biodiversity conservation. Biological Conservation 134:113-121.
