# Supplementary material for: The Impact of Land Abandonment on Species Richness and Abundance in the Mediterranean Basin: A Meta-Analysis
Source: PLoS One. 2014 May 27;9(5):e98355. doi: 10.1371/journal.pone.0098355 (PMC4035294; doi:10.1371/journal.pone.0098355)
Supplement: Table S3 — Cases included in the meta-analysis: Dependent variables. (PDF) [file pone.0098355.s004.pdf]

Table S3. Cases included in the meta-analysis: Dependent variables. Type: A = Abundance, R = Richness; Mean (Ref): Mean richness / abundance values of reference plots; SD (Ref) = Standard deviation of richness / abundance values of reference plots; N (Ref) = Number of reference plots; Mean (abandoned) = Mean richness / abundance values of abandoned plots; SD (abandoned) = Standard deviation of richness / abundance values of abandoned plots; N (abandoned) = Number of abandoned plots; Sample type: D = Dependent, I = Independent; sd_p = pooled standard deviation; ES = Effect size; SD = Standard deviation of the effect size.

ID	Reference	Kingdom	Taxon	Type	Mean (Ref)	SD (Ref)	N (Ref)	Mean (abandoned)	SD (abandoned)	N (abandoned)	Sample type	sd_p	ES	SD
1	Aragón et al. 2010	Fungi	Lichen	R	14.00	2.60	60	17.10	3.30	60	I	2.97069	1.04353	0.19460
2	Aragón et al. 2010	Fungi	Lichen	R	23.40	2.60	6	33.70	1.50	6	I	2.12250	4.85277	1.14654
3	Aragón et al. 2010	Fungi	Lichen	R	12.90	3.10	60	17.10	3.30	60	I	3.20156	1.31186	0.20126
4	Aragón et al. 2010	Fungi	Lichen	R	21.90	1.60	6	33.70	1.50	6	I	1.55081	7.60895	1.65701
5	Allen et al. 2006	Plants	Vascular	R	13.94	6.06	32	17.08	5.89	24	I	5.98818	0.52437	0.27454
6	Andres & Ojeda 2002	Plants	Vascular	R	16.10	3.10	10	11.80	2.30	10	D	2.72947	-1.57540	0.51191
7	Bonamomi et al. 2009	Plants	Vascular	R	7.80	2.60	8	4.70	1.10	9	I	1.94936	-1.59027	0.55722
8	Bonet 2004	Plants	Vascular	R	13.70	6.10	11	19.10	4.20	18	I	4.98880	1.08242	0.40825
9	Bonet 2004	Plants	Vascular	R	13.70	6.10	11	21.60	5.70	18	I	5.85134	1.35012	0.42177
10	Carmona et al. 2012	Plants	Vascular	R	6.42	4.27	8	7.00	5.26	8	I	4.79153	0.12105	0.50046
11	Carmona et al. 2012	Plants	Vascular	R	2.41	1.50	8	5.32	5.32	8	I	3.90656	0.74490	0.51705
12	Carmona et al. 2012	Plants	Vascular	R	6.14	4.13	8	5.70	3.79	8	I	3.96343	-0.11101	0.50038
13	Carmona et al. 2012	Plants	Vascular	R	6.83	3.65	8	10.92	7.86	8	I	6.12944	0.66727	0.51373
14	Castro et al. 2010	Plants	Vascular	R	10.17	3.54	45	18.67	6.00	45	I	4.92747	1.72502	0.24693

15	Castro et al. 2010	Plants	Vascular	R	10.17	3.54	45	6.40	4.48	45	I	4.04142	-0.93284	0.22199
16	Catorci 2011a	Plants	Vascular	R	15.30	6.44	50	10.50	4.95	50	I	5.74352	-0.83572	0.20855
17	Catorci 2011a	Plants	Vascular	R	25.60	8.34	50	21.70	10.82	50	I	9.65992	-0.40373	0.20203
18	Catorci 2011a	Plants	Vascular	R	19.30	7.99	50	15.00	8.13	50	I	8.06030	-0.53348	0.20353
19	Catorci 2011a	Plants	Vascular	R	12.30	4.60	50	8.30	2.62	50	I	3.74329	-1.06858	0.21380
20	Catorci 2011b	Plants	Vascular	R	45.00	3.20	19	45.70	5.90	24	D	4.90134	0.14282	0.30747
21	Celik et al. 2011	Plants	Vascular	R	14.00	0.00	1	34.00	0.00	1	D	NA	NA	NA
22	Celik et al. 2011	Plants	Vascular	R	18.00	0.00	1	34.00	0.00	1	I	NA	NA	NA
23	Curt 2003	Plants	Vascular	R	38.00	3.20	7	46.50	13.30	8	I	9.99873	0.85011	0.54032
24	de Bello et al 2006	Plants	Vascular	R	21.20	8.10	4	24.90	5.80	4	I	7.04450	0.52523	0.71920
25	de Bello et al 2006	Plants	Vascular	R	42.50	9.00	4	35.00	5.20	4	I	7.34983	-1.02043	0.75172
26	de Bello et al 2006	Plants	Vascular	R	51.50	3.00	4	32.70	6.20	4	I	4.87032	-3.86012	1.19636
27	de Bello et al 2006	Plants	Vascular	R	46.80	6.00	4	44.70	6.00	4	I	6.00000	-0.35000	0.71250
28	de Bello et al 2006	Plants	Vascular	R	40.20	3.00	4	35.00	5.80	4	I	4.61736	-1.12619	0.76110
29	de Bello et al 2006	Plants	Vascular	R	28.80	6.00	4	24.90	5.80	4	I	5.90085	-0.66092	0.72616
30	de Bello et al 2006	Plants	Vascular	R	38.30	5.80	4	35.00	5.20	4	I	5.50818	-0.59911	0.72280
31	de Bello et al 2006	Plants	Vascular	R	43.00	3.00	4	32.70	6.20	4	I	4.87032	-2.11485	0.88291
32	de Bello et al 2006	Plants	Vascular	R	46.20	3.00	4	44.70	6.00	4	I	4.74342	-0.31623	0.71151
33	de Bello et al 2006	Plants	Vascular	R	45.50	2.40	4	35.00	5.80	4	I	4.43847	-2.36568	0.92183
34	Debussche et al. 1996	Plants	Vascular	R	52.20	9.10	8	39.00	8.40	8	I	8.75700	-1.50737	0.56657
35	Farris et al. 2010	Plants	Vascular	R	12.30	2.40	10	10.20	0.40	10	I	1.72047	-1.22060	0.48708

36	Gondard et al. 2001	Plants	Vascular	R	38.40	2.76	5	21.80	2.51	5	I	2.63796	-6.29273	1.54270
37	Gondard et al. 2006	Plants	Vascular	R	53.00	3.22	5	19.00	1.61	5	I	2.54563	-13.35620	3.05277
38	Houssard et al. 1980	Plants	Vascular	R	40.00	9.50	3	55.00	6.80	9	I	7.41903	2.02183	0.78407
39	Kosic et al. 2012	Plants	Vascular	R	33.90	9.50	14	33.70	15.50	3	I	10.50000	-0.01905	0.63622
40	Kosic et al. 2012	Plants	Vascular	R	47.40	8.70	11	53.40	15.40	29	I	13.95234	0.43004	0.35736
41	Kosic et al. 2012	Plants	Vascular	R	55.00	8.40	4	48.80	11.50	12	I	10.91012	-0.56828	0.58602
42	Lesschen et al. 2008	Plants	Vascular	R	30.00	7.07	2	15.00	0.00	2	I	4.99924	-3.00045	1.45785
43	López-i-Gelats & Bartolome 2008	Plants	Vascular	R	23.60	3.10	44	25.80	9.70	112	I	8.39651	0.26201	0.17854
44	Martínez-Duro et al. 2012	Plants	Vascular	R	33.80	6.60	3	39.10	3.00	2	I	5.66039	0.93633	0.95969
45	Martínez-Duro et al. 2012	Plants	Vascular	R	33.80	6.60	3	35.10	6.40	3	I	6.50077	0.19998	0.81853
46	Martínez-Duro et al. 2012	Plants	Vascular	R	33.80	6.60	3	36.40	10.90	2	I	8.28513	0.31382	0.91825
47	Martínez-Duro et al. 2012	Plants	Vascular	R	33.80	6.60	3	42.10	0.00	1	I	6.60000	1.25758	1.23734
48	Martínez-Duro et al. 2012	Plants	Vascular	R	33.80	6.60	3	23.30	1.70	3	I	4.81923	-2.17877	1.03066
49	Martínez-Duro et al. 2012	Plants	Vascular	R	14.70	6.80	3	21.00	0.00	2	I	5.55218	1.13469	0.98086
50	Martínez-	Plants	Vascular	R	14.70	6.80	3	25.00	4.00	3	I	5.57853	1.84636	0.97507

	Duro et al. 2012													
51	Martínez-Duro et al. 2012	Plants	Vascular	R	14.70	6.80	3	26.80	6.20	2	I	6.60606	1.83165	1.08112
52	Martínez-Duro et al. 2012	Plants	Vascular	R	14.70	6.80	3	27.30	0.00	1	I	6.80000	1.85294	1.32759
53	Martínez-Duro et al. 2012	Plants	Vascular	R	14.70	6.80	3	17.00	4.50	3	I	5.76585	0.39890	0.82458
54	Martínez-Duro et al. 2012	Plants	Vascular	R	13.80	8.00	3	18.00	4.40	2	I	7.00857	0.59927	0.93233
55	Martínez-Duro et al. 2012	Plants	Vascular	R	13.80	8.00	3	19.20	4.40	3	I	6.45600	0.83643	0.85145
56	Martínez-Duro et al. 2012	Plants	Vascular	R	13.80	8.00	3	19.50	5.10	2	I	7.16496	0.79554	0.94690
57	Martínez-Duro et al. 2012	Plants	Vascular	R	13.80	8.00	3	23.10	0.00	1	I	8.00000	1.16250	1.22567
58	Martínez-Duro et al. 2012	Plants	Vascular	R	13.80	8.00	3	10.80	2.10	3	I	5.84850	-0.51295	0.82982
59	Mesléard et al. 1999	Plants	Vascular	R	7.00	0.87	3	5.30	0.52	3	I	0.71412	-2.38055	1.06720
60	Mesléard et al. 1999	Plants	Vascular	R	8.00	1.56	3	5.30	0.52	3	D	1.16186	-2.32386	1.05674
61	Ne'eman & Izhaki 1995	Plants	Vascular	R	1.90	1.10	5	2.40	1.00	5	I	1.05119	0.47565	0.64134
62	Ne'eman & Izhaki 1995	Plants	Vascular	R	0.00	0.00	5	0.70	1.10	5	I	0.77782	0.89995	0.66370
63	Ne'eman & Izhaki 1995	Plants	Vascular	R	1.90	1.10	5	4.10	1.70	5	I	1.43178	1.53655	0.71976
64	Ne'eman &	Plants	Vascular	R	0.00	0.00	5	2.30	0.90	5	I	0.63640	3.61410	1.02620

	Izhaki 1995													
65	Pala & Sinis-calco 2000	Plants	Vascular	R	20.00	4.70	4	23.80	5.70	12	I	5.50104	0.69078	0.59012
66	Pala & Sinis-calco 2000	Plants	Vascular	R	20.00	4.70	4	12.40	2.20	14	I	2.84154	-2.67460	0.72121
67	Peco et al. 2006	Plants	Vascular	R	16.60	2.82	5	13.90	1.21	5	I	2.16985	-1.24433	0.69095
68	Peco et al. 2006	Plants	Vascular	R	11.80	1.72	5	13.20	4.11	5	I	3.15044	0.44438	0.64021
69	Peco et al. 2012	Plants	Vascular	R	33.00	2.70	19	35.10	2.80	19	I	2.75045	0.76351	0.33606
70	Porto et al., 2011	Plants	Vascular	R	23.62	7.05	13	20.75	7.54	4	I	7.15069	-0.40136	0.57590
71	Porto et al., 2011	Plants	Vascular	R	23.62	7.05	13	15.58	8.35	12	I	7.69917	-1.04427	0.42669
72	Potts et al. 2006	Plants	Vascular	R	38.30	7.45	3	25.30	3.46	3	I	5.80836	-2.23815	1.04121
73	Pretto et al. 2010	Plants	Vascular	R	47.58	7.30	12	26.33	8.93	12	I	8.15582	-2.60550	0.55506
74	Puerto & Rico 1988	Plants	Vascular	R	22.30	2.70	6	63.30	3.60	6	I	3.18198	12.88506	2.69277
75	Puerto & Rico 1988	Plants	Vascular	R	22.20	2.30	6	60.80	6.60	6	I	4.94217	7.81034	1.69560
76	Puerto & Rico 1988	Plants	Vascular	R	22.30	2.70	6	67.00	6.80	6	I	5.17349	8.64020	1.85577
77	Puerto & Rico 1988	Plants	Vascular	R	22.20	2.30	6	67.30	8.30	6	I	6.09016	7.40539	1.61812
78	Redondo Prieto 1974	Plants	Vascular	R	23.00	0.00	1	25.00	7.10	2	I	7.10000	0.28169	1.23013
79	Romane & Valerino 1997	Plants	Vascular	R	28.77	4.16	12	21.70	2.81	13	I	3.52084	-2.00805	0.49082
80	Said 2001	Plants	Vascular	R	19.10	1.00	67	21.10	1.20	65	I	1.10300	1.81323	0.20679
81	Said 2001	Plants	Vascular	R	19.10	1.00	67	21.30	1.50	52	I	1.24293	1.77001	0.21753
82	Santana et al., 2011	Plants	Vascular	R	6.46	2.90	13	13.25	2.99	4	I	2.91822	2.32676	0.69725

83	Santana et al., 2011	Plants	Vascular	R	6.46	2.90	13	15.83	2.76	12	I	2.83391	3.30639	0.61555
84	Schmitz et al. 2007	Plants	Vascular	R	2.12	0.97	14	5.09	1.16	3	I	0.99743	2.97766	0.81581
85	Skornik et al. 2010	Plants	Vascular	R	12.40	2.20	15	14.60	2.10	15	I	2.15058	1.02298	0.38830
86	Skornik et al. 2010	Plants	Vascular	R	17.20	3.30	15	14.60	2.10	15	I	2.76586	-0.94003	0.38479
87	Skornik et al. 2010	Plants	Vascular	R	22.00	22.50	15	14.60	2.10	15	I	15.97905	-0.46311	0.37001
88	Tárrega et al. 2009	Plants	Vascular	R	16.38	2.14	3	12.38	6.58	3	I	4.89218	-0.81695	0.84987
89	Tárrega et al. 2009	Plants	Vascular	R	48.00	8.89	3	40.00	17.69	3	I	14.00000	-0.57143	0.83299
90	Arroyo at al. 2005	Ani-mals	Arthro-pods	R	6.50	4.80	6	13.50	6.50	2	I	5.12266	1.36648	0.88508
91	Azcarate et al. 2012	Ani-mals	Arthro-pods	R	1.38	1.59	3	3.01	0.81	3	I	1.26178	1.29182	0.89763
92	Azcarate et al. 2012	Ani-mals	Arthro-pods	R	4.00	2.66	3	5.96	3.09	3	I	2.88303	0.67984	0.83975
93	Azcarate et al. 2012	Ani-mals	Arthro-pods	R	6.56	1.82	3	8.80	1.70	3	I	1.76102	1.27199	0.89526
94	Azcarate et al. 2012	Ani-mals	Arthro-pods	R	10.41	1.59	3	10.80	1.65	3	I	1.62028	0.24070	0.81945
95	Azcarate et al. 2012	Ani-mals	Arthro-pods	R	4.96	1.58	3	5.56	1.85	3	I	1.72031	0.34878	0.82268
96	Azcarate et al. 2012	Ani-mals	Arthro-pods	R	1.60	1.13	3	2.07	1.15	3	I	1.14004	0.41226	0.82512
97	Azcarate et al. 2012	Ani-mals	Arthro-pods	R	0.36	0.87	3	1.28	1.60	3	I	1.28781	0.71439	0.84214
98	Azcarate et al. 2012	Ani-mals	Arthro-pods	R	0.78	0.83	3	2.24	1.58	3	I	1.26200	1.15689	0.88216
99	Azcarate et al. 2012	Ani-mals	Arthro-pods	R	2.37	1.73	3	6.78	2.48	3	I	2.13814	2.06254	1.01053
100	Azcarate et al. 2012	Ani-mals	Arthro-pods	R	9.05	1.81	3	8.20	1.35	3	I	1.59665	-0.53236	0.83083
101	Azcarate et	Ani-	Arthro-	R	2.34	1.25	3	3.22	2.60	3	I	2.03991	0.43139	0.82594

	al. 2012	mals	pods											
102	Azcarate et al. 2012	Animals	Arthropods	R	2.35	2.15	3	0.41	0.61	3	I	1.58028	-1.22763	0.89009
103	Azcarate et al. 2012	Animals	Arthropods	R	1.23	1.19	3	3.00	2.08	3	I	1.69448	1.04457	0.87040
104	Azcarate et al. 2012	Animals	Arthropods	R	3.61	1.70	3	6.60	1.70	3	I	1.70000	1.75882	0.96149
105	Azcarate et al. 2012	Animals	Arthropods	R	7.21	3.28	3	9.16	2.25	3	I	2.81255	0.69332	0.84067
106	Azcarate et al. 2012	Animals	Arthropods	R	8.45	0.55	3	7.57	2.14	3	I	1.56239	-0.56324	0.83253
107	Azcarate et al. 2012	Animals	Arthropods	R	5.00	1.20	3	5.59	2.47	3	I	1.94176	0.30385	0.82119
108	Azcarate et al. 2012	Animals	Arthropods	R	1.88	3.08	3	1.02	1.36	3	I	2.38076	-0.36123	0.82313
109	Barriga et al. 2010	Animals	Arthropods	R	13.10	3.70	18	11.70	4.20	23	I	3.98976	-0.35090	0.31707
110	Barriga et al. 2010	Animals	Arthropods	R	8.60	3.60	23	11.70	4.20	23	I	3.91152	0.79253	0.30624
111	Borghesio et al. 2005	Animals	Arthropods	R	2.49	1.97	22	4.64	3.42	36	I	2.96067	0.72619	0.27889
112	David et al. 1999	Animals	Arthropods	R	2.60	0.85	8	5.00	1.34	5	I	1.05485	2.27520	0.72395
113	Fadda et al. 2008	Animals	Arthropods	R	39.06	6.44	6	37.61	4.41	6	I	5.51913	-0.26272	0.57984
114	Fadda et al. 2008	Animals	Arthropods	R	27.31	4.02	6	27.24	4.41	6	I	4.21951	-0.01659	0.57736
115	Fadda et al. 2008	Animals	Arthropods	R	20.02	3.31	6	22.24	4.85	6	I	4.15202	0.53468	0.58758
116	García-Tejero et al. 2013	Animals	Arthropods	R	20.00	3.00	3	15.33	8.65	3	I	6.47389	-0.72136	0.84263
117	García-Tejero et al. 2013	Animals	Arthropods	R	20.67	7.37	3	22.00	3.61	3	I	5.80297	0.22919	0.81917
118	Gómez et al. 2003	Animals	Arthropods	R	6.17	1.47	6	9.00	3.22	6	I	2.50293	1.13068	0.62177
119	Gómez et al. 2003	Animals	Arthropods	R	6.17	1.47	6	8.17	2.93	6	I	2.31795	0.86283	0.60362

120	Gómez et al. 2003	Animals	Arthropods	R	6.17	1.47	6	11.67	2.94	6	I	2.32427	2.36633	0.75276
121	Pleixida et al. 2012	Animals	Birds	R	0.48	0.02	42	0.57	0.77	42	I	0.54466	0.16524	0.21859
122	Pleixida et al. 2012	Animals	Arthropods	R	12.07	4.55	42	12.59	5.00	42	I	4.78030	0.10878	0.21838
123	Potts et al. 2006	Animals	Arthropods	R	19.00	2.94	3	13.00	2.08	3	I	2.54657	-2.35611	1.06267
124	Santana et al., 2012	Animals	Birds	R	9.33	2.46	12	11.67	3.51	3	I	2.64877	0.88343	0.66534
125	Santana et al., 2012	Animals	Birds	R	9.33	2.46	12	12.20	2.65	15	I	2.56813	1.11754	0.41609
126	Santana et al., 2012	Animals	Birds	R	13.85	3.11	13	15.50	3.70	4	I	3.23662	0.50979	0.57842
127	Santana et al., 2012	Animals	Birds	R	13.85	3.11	13	14.35	2.44	12	I	2.80957	0.17796	0.40111
128	Scalercio et al., 2007	Animals	Arthropods	R	18.00	0.00	1	21.00	0.00	1	I	NA	NA	NA
129	Scalercio et al., 2007	Animals	Arthropods	R	61.00	0.00	1	95.00	0.00	1	I	NA	NA	NA
130	Verdasca et al., 2012	Animals	Arthropods	R	14.15	2.76	13	13.33	3.06	3	I	2.80482	-0.29235	0.64259
131	Verdasca et al., 2012	Animals	Arthropods	R	14.15	2.76	13	10.33	2.84	12	I	2.79855	-1.36499	0.44443
132	Zamora et al., 2007	Animals	Arthropods	R	10.30	1.20	3	11.70	1.20	6	I	1.20000	1.16667	0.75869
133	Zamora et al., 2007	Animals	Arthropods	R	11.50	1.30	6	8.80	1.50	4	I	1.37840	-1.95879	0.78007
134	Arroyo at al. 2005	Animals	Arthropods	A	37.80	41.60	6	126.00	29.00	2	I	39.77813	2.21730	0.98689
135	Barriga et al. 2010	Animals	Arthropods	A	12.00	7.60	18	8.20	4.40	23	I	6.00820	-0.63247	0.32235
136	Barriga et al. 2010	Animals	Arthropods	A	8.10	5.10	23	8.20	4.40	23	I	4.76288	0.02100	0.29489
137	David et al. 1999	Animals	Arthropods	A	95.00	50.91	8	90.00	40.25	5	I	47.31235	-0.10568	0.57046
138	Fadda et al.	Ani-	Arthro-	A	130.25	35.52	6	124.66	16.31	6	I	27.63771	-0.20226	0.57882

	2008	mals	pods											
139	Fadda et al. 2008	Animals	Arthropods	A	137.88	38.75	6	95.65	17.00	6	I	29.92125	-1.41137	0.64524
140	Fadda et al. 2008	Animals	Arthropods	A	130.25	36.74	6	238.44	93.08	6	I	70.75915	1.52899	0.65631
141	García-Tejero et al. 2013	Animals	Arthropods	A	357.66	162.54	3	123.33	98.90	3	I	134.53710	-1.74175	0.95889
142	García-Tejero et al. 2013	Animals	Arthropods	A	134.00	107.85	3	81.67	9.29	3	I	76.54387	-0.68366	0.84001
143	Gómez et al. 2003	Animals	Arthropods	A	171.83	152.43	6	218.00	150.24	6	I	151.33896	0.30508	0.58070
144	Gómez et al. 2003	Animals	Arthropods	A	171.83	152.43	6	91.00	31.96	6	I	110.12799	-0.73396	0.59647
145	Gómez et al. 2003	Animals	Arthropods	A	171.83	152.43	6	148.50	63.01	6	I	116.63011	-0.20003	0.57879
146	Pleixida et al. 2012	Animals	Birds	A	0.58	0.07	42	0.86	0.07	42	I	0.07000	4.00000	0.37796
147	Pleixida et al. 2012	Animals	Arthropods	A	102.16	110.67	42	48.05	56.89	42	I	87.98955	-0.61496	0.22332
148	Potts et al. 2006	Animals	Arthropods	A	231.00	5.10	3	122.00	31.18	3	I	22.34057	-4.87902	1.62800
149	Santana et al., 2012	Animals	Birds	A	16.58	4.96	12	18.33	4.11	3	I	4.83896	0.36165	0.64887
150	Santana et al., 2012	Animals	Birds	A	16.58	4.96	12	22.53	6.44	15	I	5.83523	1.01967	0.41141
151	Santana et al., 2012	Animals	Birds	A	19.38	4.11	13	21.00	2.83	4	I	3.88786	0.41668	0.57622
152	Santana et al., 2012	Animals	Birds	A	19.38	4.11	13	19.67	5.18	12	I	4.65254	0.06233	0.40042
153	Verdasca et al., 2012	Animals	Arthropods	A	70.15	28.96	13	46.67	2.52	3	I	26.82866	-0.87518	0.65893
154	Verdasca et al., 2012	Animals	Arthropods	A	70.15	28.96	13	30.83	11.83	12	I	22.46119	-1.75058	0.47069
