# Supplementary material for: The Impact of Land Abandonment on Species Richness and Abundance in the Mediterranean Basin: A Meta-Analysis
Source: PLoS One. 2014 May 27;9(5):e98355. doi: 10.1371/journal.pone.0098355 (PMC4035294; doi:10.1371/journal.pone.0098355)
Supplement: Table S4 — Cases included in the meta-analysis: Independent variables. (PDF) [file pone.0098355.s005.pdf]

Table S4. Cases included in the meta-analysis: Independent variables. TSA = Time since abandonment; Mountain area: M = Mountain area, L = Lowland area.

ID	Reference	Unit size (m²)	Extent class (km²)	TSA (years)	Previous land use	Ecological region	Country	Landform	Protected area status	Temperature (°C)	Precipitation (mm)
1	Aragón et al. 2010	<1	100>10000	30-49	agroforestry	Iberian sclerophyllous and semi-deciduous forests	Spain	M	Yes	12.8	500
2	Aragón et al. 2010	>100	100>10000	30-49	agroforestry	Iberian sclerophyllous and semi-deciduous forests	Spain	M	Yes	12.8	500
3	Aragón et al. 2010	<1	100>10000	30-49	agroforestry	Iberian sclerophyllous and semi-deciduous forests	Spain	M	Yes	12.8	500
4	Aragón et al. 2010	>100	100>10000	30-49	agroforestry	Iberian sclerophyllous and semi-deciduous forests	Spain	M	Yes	12.8	500
5	Allen et al. 2006	10<100	100>10000	5-9	permanent crops	Crete Mediterranean Forests	Greece	M	No	16.6	926
6	Andres & Ojeda 2002	>100	100>10000	30-49	pastures	Southwest Iberian Mediterranean sclerophyllous and mixed forests	Spain	M	Yes	16.8	825
7	Bonamomi et al. 2009	<1	1>100	10-29	pastures	Italian sclerophyllous and semi-deciduous forests	Italy	M	No	11	949
8	Bonet 2004	10<100	1>100	30-49	permanent crops	Iberian sclerophyllous and semi-deciduous forests	Spain	M	No	15.2	457
9	Bonet 2004	10<100	1>100	5-9	permanent crops	Iberian sclerophyllous and semi-deciduous forests	Spain	M	No	15.2	457
10	Carmona et al. 2012	<1	1>100	30-49	agroforestry	Iberian sclerophyllous and semi-deciduous forests	Spain	M	Yes	11.9	479
11	Carmona et al. 2012	<1	1>100	30-49	agroforestry	Iberian sclerophyllous and semi-deciduous forests	Spain	M	Yes	11.9	479
12	Carmona et al. 2012	<1	1>100	30-49	agroforestry	Iberian sclerophyllous and semi-deciduous forests	Spain	M	Yes	11.9	479
13	Carmona et al. 2012	<1	1>100	30-49	agroforestry	Iberian sclerophyllous and semi-deciduous forests	Spain	M	Yes	11.9	479
14	Castro et al. 2010	1<10	1>100	10-29	agroforestry	Iberian sclerophyllous and semi-deciduous forests	Portugal	L	Yes	16.7	529
15	Castro et al.	1<10	1>100	10-29	agroforestry	Iberian sclerophyllous and semi-	Portugal	L	Yes	16.7	529

	2010					deciduous forests					
16	Catorci 2011a	1<10	100>10000	10-29	pastures	Italian sclerophyllous and semi-deciduous forests	Italy	M	No	7.4	891
17	Catorci 2011a	1<10	100>10000	10-29	pastures	Italian sclerophyllous and semi-deciduous forests	Italy	M	No	7.4	891
18	Catorci 2011a	1<10	100>10000	10-29	pastures	Italian sclerophyllous and semi-deciduous forests	Italy	M	No	7.4	891
19	Catorci 2011a	1<10	100>10000	10-29	pastures	Italian sclerophyllous and semi-deciduous forests	Italy	M	No	7.4	891
20	Catorci 2011b	10<100	100>10000	10-29	pastures	Italian sclerophyllous and semi-deciduous forests	Italy	M	No	7	893
21	Celik et al. 2011	10<100	<1	10-29	arable land	Euxine-Colchic deciduous forest	Turkey	L		13.6	1382
22	Celik et al. 2011	10<100	<1	10-29	arable land	Euxine-Colchic deciduous forest	Turkey	L		13.6	1382
23	Curt 2003	10<100	1>100	30-49	pastures	Western European broadleaf forests	France	M	No	8	800
24	de Bello et al 2006a	10<100	1>100	10-29	pastures	Iberian sclerophyllous and semi-deciduous forests	Spain	L	Yes	14.8	358
25	de Bello et al 2006a	10<100	1>100	10-29	pastures	Iberian sclerophyllous and semi-deciduous forests	Spain	M	Yes	14.1	512
26	de Bello et al 2006a	10<100	1>100	10-29	pastures	Iberian sclerophyllous and semi-deciduous forests	Spain	M	No	12.2	638
27	de Bello et al 2006a	10<100	1>100	10-29	pastures	Pyrenees conifer and mixed forests	Spain	M	No	9.1	914
28	de Bello et al 2006a	10<100	1>100	10-29	pastures	Pyrenees conifer and mixed forests	Spain	M	Yes	6.5	1078
29	de Bello et al 2006a	10<100	1>100	10-29	pastures	Iberian sclerophyllous and semi-deciduous forests	Spain	L	Yes	14.8	358
30	de Bello et al 2006a	10<100	1>100	10-29	pastures	Iberian sclerophyllous and semi-deciduous forests	Spain	M	Yes	14.1	512
31	de Bello et al 2006a	10<100	1>100	10-29	pastures	Iberian sclerophyllous and semi-deciduous forests	Spain	M	No	12.2	638
32	de Bello et al 2006a	10<100	1>100	10-29	pastures	Pyrenees conifer and mixed forests	Spain	M	No	9.1	914
33	de Bello et al 2006a	10<100	1>100	10-29	pastures	Pyrenees conifer and mixed forests	Spain	M	Yes	6.5	1078

34	Debussche et al. 1996		1>100	≥50	permanent crops	Northeastern Spain & Southern France Mediterranean	France	M	Yes	13	733
35	Farris et al. 2010	<1	100>10000	5-9	pastures	Tyrrhenian-Adriatic sclerophyllous and mixed forests	Italy	M	Yes	12.5	873
36	Gondard et al. 2001	10<100	1>100	5-9	permanent crops	Western European broadleaf forests	France	M	Yes	10.3	758
37	Gondard et al. 2006	10<100	1>100	5-9	permanent crops	Northwest Iberian montane forests	Spain	M	Yes	11.2	574
38	Houssard et al. 1980		1>100	10-29	permanent crops	Northwest Iberian montane forests	France	M	Yes	13	731
39	Kosic et al. 2012	10<100	1>100	30-49	pastures	Dinaric Mountains mixed forests	Croatia	M	Yes	10.6	1271
40	Kosic et al. 2012	10<100	1>100	30-49	pastures	Dinaric Mountains mixed forests	Croatia	M	Yes	10.6	1271
41	Kosic et al. 2012	10<100	1>100	30-49	pastures	Dinaric Mountains mixed forests	Croatia	M	Yes	10.6	1271
42	Lesschen et al. 2008	10<100	1>100	≥50	arable land	Iberian sclerophyllous and semi-deciduous forests	Spain	M	Yes	16.4	331
43	López-i-Gelats & Bartolome 2008	>100	100>10000	10-29	pastures	Pyrenees conifer and mixed forests	Spain	M	Yes	7.8	1023
44	Martínez-Duro et al. 2012	10<100	1>100	5-9	permanent crops	Iberian sclerophyllous and semi-deciduous forests	Spain	L	Yes	13.5	443
45	Martínez-Duro et al. 2012	10<100	1>100	10-29	permanent crops	Iberian sclerophyllous and semi-deciduous forests	Spain	L	Yes	13.5	443
46	Martínez-Duro et al. 2012	10<100	1>100	10-29	permanent crops	Iberian sclerophyllous and semi-deciduous forests	Spain	L	Yes	13.5	443
47	Martínez-Duro et al. 2012	10<100	1>100	30-49	permanent crops	Iberian sclerophyllous and semi-deciduous forests	Spain	L	Yes	13.5	443
48	Martínez-Duro et al. 2012	10<100	1>100	≥50	permanent crops	Iberian sclerophyllous and semi-deciduous forests	Spain	L	Yes	13.5	443

49	Martínez-Duro et al. 2012	10<100	1>100	5-9	permanent crops	Iberian sclerophyllous and semi-deciduous forests	Spain	L	Yes	13.5	443
50	Martínez-Duro et al. 2012	10<100	1>100	10-29	permanent crops	Iberian sclerophyllous and semi-deciduous forests	Spain	L	Yes	13.5	443
51	Martínez-Duro et al. 2012	10<100	1>100	10-29	permanent crops	Iberian sclerophyllous and semi-deciduous forests	Spain	L	Yes	13.5	443
52	Martínez-Duro et al. 2012	10<100	1>100	30-49	permanent crops	Iberian sclerophyllous and semi-deciduous forests	Spain	L	Yes	13.5	443
53	Martínez-Duro et al. 2012	10<100	1>100	≥50	permanent crops	Iberian sclerophyllous and semi-deciduous forests	Spain	L	Yes	13.5	443
54	Martínez-Duro et al. 2012	10<100	1>100	5-9	permanent crops	Iberian sclerophyllous and semi-deciduous forests	Spain	L	Yes	13.5	443
55	Martínez-Duro et al. 2012	10<100	1>100	10-29	permanent crops	Iberian sclerophyllous and semi-deciduous forests	Spain	L	Yes	13.5	443
56	Martínez-Duro et al. 2012	10<100	1>100	10-29	permanent crops	Iberian sclerophyllous and semi-deciduous forests	Spain	L	Yes	13.5	443
57	Martínez-Duro et al. 2012	10<100	1>100	30-49	permanent crops	Iberian sclerophyllous and semi-deciduous forests	Spain	L	Yes	13.5	443
58	Martínez-Duro et al. 2012	10<100	1>100	≥50	permanent crops	Iberian sclerophyllous and semi-deciduous forests	Spain	L	Yes	13.5	443
59	Mesléard et al. 1999	1<10	<1	5-9	pastures	Northeastern Spain & Southern France Mediterranean	France	L	Yes	13.8	670
60	Mesléard et al. 1999	1<10	<1	5-9	pastures	Northeastern Spain & Southern France Mediterranean	France	L	Yes	13.8	670
61	Ne'eman & Izhaki 1995	1<10	<1	10-29	permanent crops	Middle East steppe	Israel	L		17.3	427
62	Ne'eman &	1<10	<1	10-29	permanent	Middle East steppe	Israel	L		17.3	427

	Izhaki 1995				crops						
63	Ne'eman & Izhaki 1995	1<10	<1	30-49	permanent crops	Middle East steppe	Israel	L		17.3	427
64	Ne'eman & Izhaki 1995	1<10	<1	30-49	permanent crops	Middle East steppe	Israel	L		17.3	427
65	Pala & Sinis-calco 2000	10<100	1>100	5-9	pastures	Appenine deciduous montane forests	Italy	M	No	9.7	1065
66	Pala & Sinis-calco 2000	10<100	1>100	10-29	pastures	Appenine deciduous montane forests	Italy	M	No	9.7	1065
67	Peco et al. 2006	<1	100>10000	30-49	agroforestry	Iberian sclerophyllous and semi-deciduous forests	Spain	M	Yes	12.2	471
68	Peco et al. 2006	<1	100>10000	30-49	agroforestry	Iberian sclerophyllous and semi-deciduous forests	Spain	M	Yes	12.2	471
69	Peco et al. 2012	10<100	100>10000	30-49	agroforestry	Iberian sclerophyllous and semi-deciduous forests	Spain	M	Yes	12.2	466
70	Porto et al., 2011		100>10000	10-29	agroforestry	Southwest Iberian Mediterrane-an sclerophyllous and mixed forests	Portugal	M	Yes	14.9	630
71	Porto et al., 2011		100>10000	≥50	agroforestry	Southwest Iberian Mediterrane-an sclerophyllous and mixed forests	Portugal	M	Yes	14.9	630
72	Potts et al. 2006	10<100	1>100	10-29	permanent crops	Aegean & West Turkey scle-rophyllous and mixed forest	Greece	M	Yes	16.4	636
73	Pretto et al. 2010	10<100	1>100		pastures	Tyrrhenian-Adriatic scle-rophyllous and mixed forests	Italy	L	Yes	16.6	676
74	Puerto & Rico 1988	<1		10-29	agroforestry	Iberian sclerophyllous and semi-deciduous forests	Spain	L		11.5	456
75	Puerto & Rico 1988	<1		10-29	agroforestry	Iberian sclerophyllous and semi-deciduous forests	Spain	L		11.5	456
76	Puerto & Rico 1988	<1		10-29	agroforestry	Iberian sclerophyllous and semi-deciduous forests	Spain	L		11.5	456
77	Puerto & Rico 1988	<1		10-29	agroforestry	Iberian sclerophyllous and semi-deciduous forests	Spain	L		11.5	456
78	Redondo Prieto 1974	<1	<1	10-29	arable land	Iberian sclerophyllous and semi-deciduous forests	Spain	L	Yes	11.9	409
79	Romane & Valerino	10<100	1>100	5-9	permanent crops	Western European broadleaf forests	France	M	Yes	9.5	802

	1997										
80	Said 2001	10<100	1>100	10-29	pastures	Corsican montane broadleaf and mixed forests	France	M	Yes	12.7	693
81	Said 2001	10<100	1>100	30-49	pastures	Corsican montane broadleaf and mixed forests	France	M	Yes	12.7	693
82	Santana et al., 2011		100>10000	10-29	agroforestry	Southwest Iberian Mediterrane-an sclerophyllous and mixed forests	Portugal	M	Yes	14.9	630
83	Santana et al., 2011		100>10000	≥50	agroforestry	Southwest Iberian Mediterrane-an sclerophyllous and mixed forests	Portugal	M	Yes	14.9	630
84	Schmitz et al. 2007	>100	1>100		agroforestry	Iberian sclerophyllous and semi-deciduous forests	Spain	M	Yes	11.1	501
85	Skornik et al. 2010	<1	1>100	10-29	pastures	Dinaric Mountains mixed fo-rests	Slovenia	M	Yes	10.5	1324
86	Skornik et al. 2010	<1	1>100	10-29	pastures	Dinaric Mountains mixed fo-rests	Slovenia	M	Yes	10.5	1324
87	Skornik et al. 2010	<1	1>100	10-29	pastures	Dinaric Mountains mixed fo-rests	Slovenia	M	Yes	10.5	1324
88	Tárrega et al. 2009	1<10	100>10000	10-29	agroforestry	Northwest Iberian montane fo-rests	Spain	M	Yes	10	673
89	Tárrega et al. 2009	10<100	100>10000	10-29	agroforestry	Northwest Iberian montane fo-rests	Spain	M	Yes	10	673
90	Arroyo at al. 2005		1>100	5-9	arable land	Northwest Iberian montane fo-rests	Spain	L	No	10.8	568
91	Azcarate et al. 2012		1>100	30-49	agroforestry	Iberian sclerophyllous and semi-deciduous forests	Spain	M	No	12.8	418
92	Azcarate et al. 2012		1>100	30-49	agroforestry	Iberian sclerophyllous and semi-deciduous forests	Spain	M	No	12.8	418
93	Azcarate et al. 2012		1>100	30-49	agroforestry	Iberian sclerophyllous and semi-deciduous forests	Spain	M	No	12.8	418
94	Azcarate et al. 2012		1>100	30-49	agroforestry	Iberian sclerophyllous and semi-deciduous forests	Spain	M	No	12.8	418
95	Azcarate et al. 2012		1>100	30-49	agroforestry	Iberian sclerophyllous and semi-deciduous forests	Spain	M	No	12.8	418
96	Azcarate et al. 2012		1>100	30-49	agroforestry	Iberian sclerophyllous and semi-deciduous forests	Spain	M	No	12.8	418

97	Azcarate et al. 2012	1>100	30-49	agroforestry	Iberian sclerophyllous and semi-deciduous forests	Spain	M	No	12.8	418
98	Azcarate et al. 2012	1>100	30-49	agroforestry	Iberian sclerophyllous and semi-deciduous forests	Spain	M	No	12.8	418
99	Azcarate et al. 2012	1>100	30-49	agroforestry	Iberian sclerophyllous and semi-deciduous forests	Spain	M	No	12.8	418
100	Azcarate et al. 2012	1>100	30-49	agroforestry	Iberian sclerophyllous and semi-deciduous forests	Spain	M	No	12.8	418
101	Azcarate et al. 2012	1>100	30-49	agroforestry	Iberian sclerophyllous and semi-deciduous forests	Spain	M	No	12.8	418
102	Azcarate et al. 2012	1>100	30-49	agroforestry	Iberian sclerophyllous and semi-deciduous forests	Spain	M	No	12.8	418
103	Azcarate et al. 2012	1>100	30-49	agroforestry	Iberian sclerophyllous and semi-deciduous forests	Spain	M	No	12.8	418
104	Azcarate et al. 2012	1>100	30-49	agroforestry	Iberian sclerophyllous and semi-deciduous forests	Spain	M	No	12.8	418
105	Azcarate et al. 2012	1>100	30-49	agroforestry	Iberian sclerophyllous and semi-deciduous forests	Spain	M	No	12.8	418
106	Azcarate et al. 2012	1>100	30-49	agroforestry	Iberian sclerophyllous and semi-deciduous forests	Spain	M	No	12.8	418
107	Azcarate et al. 2012	1>100	30-49	agroforestry	Iberian sclerophyllous and semi-deciduous forests	Spain	M	No	12.8	418
108	Azcarate et al. 2012	1>100	30-49	agroforestry	Iberian sclerophyllous and semi-deciduous forests	Spain	M	No	12.8	418
109	Barriga et al. 2010	100>10000		agroforestry	Iberian sclerophyllous and semi-deciduous forests	Spain	M	Yes	12.7	501
110	Barriga et al. 2010	100>10000		agroforestry	Iberian sclerophyllous and semi-deciduous forests	Spain	M	Yes	12.7	501
111	Borghesio et al. 2005	1>100	10-29	arable land	Italian sclerophyllous and semi-deciduous forests	Italy	M	No	11.5	1046
112	David et al. 1999	1>100	10-29	arable land	Northeastern Spain & Southern France Mediterranean	France	M	Yes	12.9	745
113	Fadda et al. 2008	1>100	10-29	pastures	Northeastern Spain & Southern France Mediterranean	France	L	Yes	13.8	630
114	Fadda et al. 2008	1>100	10-29	pastures	Northeastern Spain & Southern France Mediterranean	France	L	Yes	13.8	630
115	Fadda et al.	1>100	5-9	pastures	Northeastern Spain & Southern	France	L	Yes	13.8	630

	2008				France Mediterranean					
116	García-Tejero et al. 2013	100>10000	10-29	agroforestry	Northwest Iberian montane forests	Spain	M	Yes	9.9	713
117	García-Tejero et al. 2013	100>10000	10-29	agroforestry	Northwest Iberian montane forests	Spain	M	Yes	9.9	713
118	Gómez et al. 2003	100>10000	10-29	arable land	Northeastern Spain & Southern France Mediterranean	Spain	L	Yes	15.3	607
119	Gómez et al. 2003	100>10000	10-29	arable land	Northeastern Spain & Southern France Mediterranean	Spain	L	Yes	15.3	607
120	Gómez et al. 2003	100>10000	30-49	arable land	Northeastern Spain & Southern France Mediterranean	Spain	L	Yes	15.3	607
121	Pleixida et al. 2012	1>100	10-29	arable land	Pindus Mountains mixed forests	Greece	M	Yes	12.3	693
122	Pleixida et al. 2012	1>100	10-29	arable land	Pindus Mountains mixed forests	Greece	M	Yes	12.3	693
123	Potts et al. 2006	1>100	10-29	permanent crops	Aegean & West Turkey sclerophyllous and mixed forest	Greece	M	Yes	16.4	636
124	Santana et al., 2012	100>10000	10-29	agroforestry	Southwest Iberian Mediterranean sclerophyllous and mixed forests	Portugal	M	Yes	14.9	630
125	Santana et al., 2012	100>10000	≥50	agroforestry	Southwest Iberian Mediterranean sclerophyllous and mixed forests	Portugal	M	Yes	14.9	630
126	Santana et al., 2012	100>10000	10-29	agroforestry	Southwest Iberian Mediterranean sclerophyllous and mixed forests	Portugal	M	Yes	14.9	630
127	Santana et al., 2012	100>10000	≥50	agroforestry	Southwest Iberian Mediterranean sclerophyllous and mixed forests	Portugal	M	Yes	14.9	630
128	Scalercio et al., 2007	<1		permanent crops	Tyrrhenian-Adriatic sclerophyllous and mixed forests	Italy	M	Yes	17.8	833
129	Scalercio et al., 2007	<1		permanent crops	Tyrrhenian-Adriatic sclerophyllous and mixed forests	Italy	M	Yes	17.8	833
130	Verdasca et al., 2012	100>10000	10-29	agroforestry	Southwest Iberian Mediterranean sclerophyllous and mixed forests	Portugal	M	Yes	14.9	630
131	Verdasca et	100>10000	≥50	agroforestry	Southwest Iberian Mediterrane-	Portugal	M	Yes	14.9	630

	al., 2012				an sclerophyllous and mixed forests					
132	Zamora et al., 2007	1>100		pastures	Iberian sclerophyllous and semi-deciduous forests	Spain	M	Yes	13.1	568
133	Zamora et al., 2007	1>100		pastures	Iberian sclerophyllous and semi-deciduous forests	Spain	M	Yes	11.6	630
134	Arroyo at al. 2005	1>100	5-9	arable land	Northwest Iberian montane forests	Spain	L	No	10.8	568
135	Barriga et al. 2010	100>10000		agroforestry	Iberian sclerophyllous and semi-deciduous forests	Spain	M	Yes	12.7	501
136	Barriga et al. 2010	100>10000		agroforestry	Iberian sclerophyllous and semi-deciduous forests	Spain	M	Yes	12.7	501
137	David et al. 1999	1>100	10-29	arable land	Northeastern Spain & Southern France Mediterranean	France	M	Yes	12.9	745
138	Fadda et al. 2008	1>100	10-29	pastures	Northeastern Spain & Southern France Mediterranean	France	L	Yes	13.8	630
139	Fadda et al. 2008	1>100	10-29	pastures	Northeastern Spain & Southern France Mediterranean	France	L	Yes	13.8	630
140	Fadda et al. 2008	1>100	5-9	pastures	Northeastern Spain & Southern France Mediterranean	France	L	Yes	13.8	630
141	García-Tejero et al. 2013	100>10000	10-29	agroforestry	Northwest Iberian montane forests	Spain	M	Yes	9.9	713
142	García-Tejero et al. 2013	100>10000	10-29	agroforestry	Northwest Iberian montane forests	Spain	M	Yes	9.9	713
143	Gómez et al. 2003	100>10000	10-29	arable land	Northeastern Spain & Southern France Mediterranean	Spain	L	Yes	15.3	607
144	Gómez et al. 2003	100>10000	10-29	arable land	Northeastern Spain & Southern France Mediterranean	Spain	L	Yes	15.3	607
145	Gómez et al. 2003	100>10000	30-49	arable land	Northeastern Spain & Southern France Mediterranean	Spain	L	Yes	15.3	607
146	Pleixida et al. 2012	1>100	10-29	arable land	Pindus Mountains mixed forests	Greece	M	Yes	12.3	693
147	Pleixida et al. 2012	1>100	10-29	arable land	Pindus Mountains mixed forests	Greece	M	Yes	12.3	693
148	Potts et al. 2006	1>100	10-29	permanent crops	Aegean & West Turkey sclerophyllous and mixed forest	Greece	M	Yes	16.4	636
149	Santana et al.,	100>10000	10-29	agroforestry	Southwest Iberian Mediterrane-	Portugal	M	Yes	14.9	630

	2012				an sclerophyllous and mixed forests					
150	Santana et al., 2012	100>10000	≥50	agroforestry	Southwest Iberian Mediterranean sclerophyllous and mixed forests	Portugal	M	Yes	14.9	630
151	Santana et al., 2012	100>10000	10-29	agroforestry	Southwest Iberian Mediterranean sclerophyllous and mixed forests	Portugal	M	Yes	14.9	630
152	Santana et al., 2012	100>10000	≥50	agroforestry	Southwest Iberian Mediterranean sclerophyllous and mixed forests	Portugal	M	Yes	14.9	630
153	Verdasca et al., 2012	100>10000	10-29	agroforestry	Southwest Iberian Mediterranean sclerophyllous and mixed forests	Portugal	M	Yes	14.9	630
154	Verdasca et al., 2012	100>10000	≥50	agroforestry	Southwest Iberian Mediterranean sclerophyllous and mixed forests	Portugal	M	Yes	14.9	630
