# Supplementary material for: The Impact of Land Abandonment on Species Richness and Abundance in the Mediterranean Basin: A Meta-Analysis
Source: PLoS One. 2014 May 27;9(5):e98355. doi: 10.1371/journal.pone.0098355 (PMC4035294; doi:10.1371/journal.pone.0098355)
Supplement: Table S5 — Summary of additional variables included in the meta-analysis. (PDF) [file pone.0098355.s006.pdf]

Table S5. Summary of additional variables included in the meta-analysis. Q = Q-test for heterogeneity (including P value); ES = effect size point estimate; SE = standard error; Z = two-tail Z-test. Categorical moderators are analyzed using mixed effect models.

**Moderator (Q, P)**	**ES**	**SE**	**Z**	**P**
Ecological region (41.91, 0.0001)				
Aegean & West Turkey sclerophyllous and mixed forest	-2.9187	1.0089	-2.8930	0.0038
Corsican montane broadleaf and mixed forests	1.7917	0.9001	1.9906	0.0465
Dinaric Mountains mixed forests	-0.0824	0.5458	-0.1509	0.8800
Iberian sclerophyllous and semi-deciduous forests	0.6582	0.1845	3.5674	0.0004
Middle East steppe	1.5046	0.7337	2.0507	0.0403
Northwest Iberian montane forests	-0.1697	0.5023	-0.3378	0.7355
Pyrenees conifer and mixed forests	-0.6730	0.6377	-1.0555	0.2912
Tyrrhenian-Adriatic sclerophyllous and mixed forests	-1.8998	0.9610	-1.9768	0.0481
Appenine deciduous montane forests	-0.9199	1.0014	-0.9186	0.3583
Italian sclerophyllous and semi-deciduous forests	-0.4930	0.4876	-1.0112	0.3119
Northeastern Spain & Southern France Mediterranean	0.0444	0.3447	0.1287	0.8976
Pindus Mountains mixed forests	0.8720	0.6415	1.3592	0.1741
Southwest Iberian Mediterranean sclerophyllous and mixed forests	-0.0110	0.3138	-0.0350	0.9720
Western European broadleaf forests	-1.6739	0.8642	-1.9370	0.0527
Country (14.33, 0.0736)				
Croatia	-0.0358	0.8473	-0.0423	0.9663
France	-0.0874	0.3700	-0.2362	0.8133
Greece	-0.1564	0.5368	-0.2913	0.7708
Israel	1.5182	0.7825	1.9402	0.0524
Italy	-0.8096	0.4312	-1.8777	0.0604
Portugal	0.1154	0.3271	0.3528	0.7243
Slovenia	-0.1279	0.8193	-0.1561	0.8760
Spain	0.4506	0.1735	2.5973	0.0094
Study (369.88, 0.0001)				
Allen et al. 2006	0.524366	0.853587	0.614309	0.539011
Andres & Ojeda 2002	-2.09976	1.282145	-1.6377	0.101485
Aragón et al. 2010	1.671813	0.999777	1.672186	0.094488
Arroyo at al. 2005	1.241061	1.221308	1.016173	0.309547
Azcarate et al. 2012	-0.01612	0.897829	-0.01795	0.985679
Barriga et al. 2010	-0.56401	0.957068	-0.58931	0.555655
Bonamomi et al. 2009	-2.11463	1.3009	-1.62552	0.104053
Bonet 2004	0.690995	1.06834	0.646793	0.517766
Borghesio et al. 2005	0.20182	1.20815	0.167049	0.867332
Carmona et al. 2012	-0.17178	0.977962	-0.17565	0.86057

Castro et al. 2010	-0.13925	1.040562	-0.13382	0.893546
Catorci 2011a	-1.23403	0.95007	-1.29889	0.193983
Catorci 2011b	-0.38155	1.215065	-0.31401	0.75351
Curt 2003	0.325742	1.293752	0.251781	0.801211
David et al. 1999	0.450697	1.123848	0.40103	0.688398
de Bello et al 2006a	-1.53688	0.926081	-1.65956	0.097003
Debussche et al. 1996	-2.03173	1.304934	-1.55696	0.11948
Fadda et al. 2008	-0.50071	0.947756	-0.52831	0.597284
Farris et al. 2010	-1.74497	1.272437	-1.37136	0.170263
García-Tejero et al. 2013	-1.21191	1.038369	-1.16713	0.243158
Gómez et al. 2003	0.038799	0.949588	0.040859	0.967408
Gondard et al. 2001	-6.8171	1.939529	-3.51482	0.00044
Gondard et al. 2006	-13.8806	3.271278	-4.24316	2.2E-05
Houssard et al. 1980	1.497462	1.413017	1.059762	0.289253
Kosic et al. 2012	-0.53599	1.018971	-0.52601	0.598883
Lesschen et al. 2008	-3.52482	1.872749	-1.88216	0.059814
López-i-Gelats & Bartolome 2008	-0.26235	1.189001	-0.22065	0.825365
Martínez-Duro et al. 2012	0.105974	0.914455	0.115888	0.907741
Mesléard et al. 1999	-2.87639	1.272437	-2.26054	0.023788
Ne'eman & Izhaki 1995	0.902558	1.015992	0.888351	0.374352
Pala & Siniscalco 2000	-1.38328	1.126467	-1.22799	0.219453
Peco et al. 2006	-0.89834	1.129837	-0.79511	0.426552
Peco et al. 2012	0.239144	1.222613	0.195601	0.844923
Pleixida et al. 2012	0.296524	0.95362	0.310946	0.755842
Porto et al., 2011	-1.2736	1.086548	-1.17215	0.241136
Potts et al. 2006	-3.3639	1.193062	-2.81955	0.004809
Pretto et al. 2010	-3.12987	1.299979	-2.40763	0.016056
Puerto & Rico 1988	8.037054	1.33065	6.039946	1.54E-09
Redondo Prieto 1974	-0.24268	1.701492	-0.14263	0.886586
Romane & Valerino 1997	-2.53241	1.273872	-1.98796	0.046816
Said 2001	1.267327	1.038146	1.22076	0.222177
Santana et al., 2011	2.3164	1.127017	2.055337	0.039846
Santana et al., 2012	0.043264	0.918065	0.047125	0.962414
Schmitz et al. 2007	2.453296	1.43087	1.714549	0.086428
Skornik et al. 2010	-0.65395	0.997375	-0.65567	0.512035
Tárrega et al. 2009	-1.21727	1.187095	-1.02542	0.305164
Verdasca et al., 2012	-1.725	0.942813	-1.82964	0.067304
Zamora et al., 2007	-0.89979	1.162391	-0.77408	0.438881
